# Supplementary material for: Mosaic RBD nanoparticles protect against challenge by diverse sarbecoviruses in animal models
Source: Science. 2022 Jul 5:eabq0839. doi: 10.1126/science.abq0839 (PMC9273039; doi:10.1126/science.abq0839)
Supplement: Supplementary file 1 — Materials and Methods Figs. S1 to S10 Data S1 to S3 [file science.abq0839_sm.pdf]

Science
AAAS

Supplementary Materials for

Mosaic RBD nanoparticles protect against challenge by diverse sarbecoviruses in animal models

Alexander A. Cohen *et al.*

Corresponding author: Pamela J. Bjorkman, bjorkman@caltech.edu

DOI: 10.1126/science.abq0839

The PDF file includes:

Figs. S1 to S10

Other Supplementary Material for this manuscript includes the following:

MDAR Reproducibility Checklist
Data S1 to S3

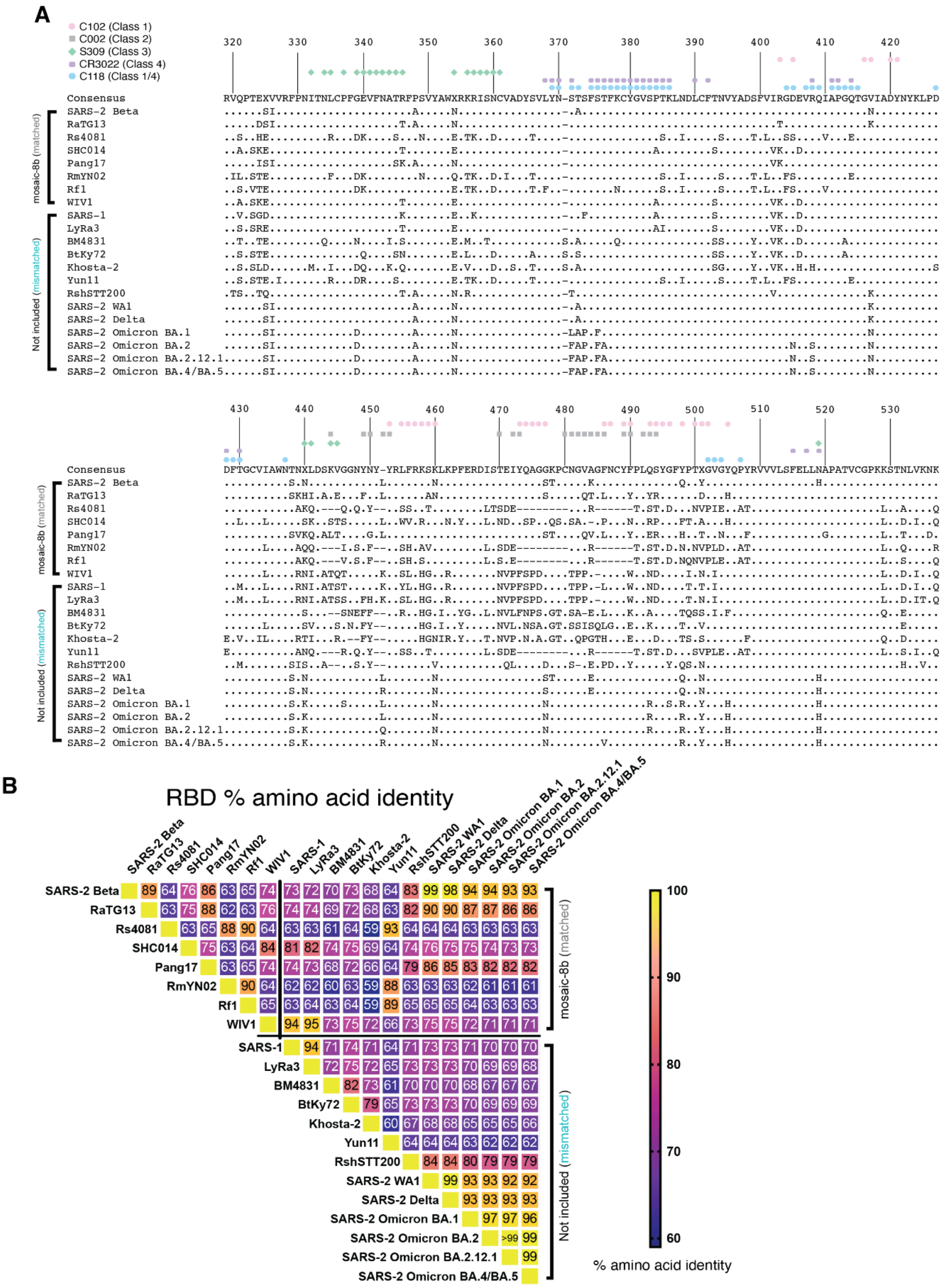
A
C102 (Class 1)
C002 (Class 2)
S309 (Class 3)
CR3022 (Class 4)
C118 (Class 1/4)
Consensus
SARS-2 Beta
RaTG13
Rs4081
SHC014
Pang17
RmYN02
Rf1
WIV1
SARS-1
LyRa3
BM4831
BtKy72
Khosta-2
Yun11
RshSTT200
SARS-2 WA1
SARS-2 Delta
SARS-2 Omicron BA.1
SARS-2 Omicron BA.2
SARS-2 Omicron BA.2.12.1
SARS-2 Omicron BA.4/BA.5
mosaic-8b (matched)
Not included (mismatched)
B
RBD % amino acid identity
mosaic-8b (matched)
Not included (mismatched)
% amino acid identity

Figure S1. Sequence alignment and identity table for RBDs from sarbecoviruses. RBDs that are present on mosaic-8b RBD-mi3 are labeled as matched, while RBDs that are not present are labeled as mismatched. **(A)** Sequence alignment of RBDs from sarbecovirus strains and SARS-2 VOCs aligned using Clustal Omega 1.2.3. RBD residues that interact with the indicated representative class 1, 2, 3, 4, and 1/4 anti-RBD antibodies (defined as a residue including one or more atoms within 4 Å of the V_H-V_L region of the bound antibody) are denoted with different colored symbols. Structures used for these assignments: C102: PDB 7K8M; C002: PDB 7K8T, S309: PDB 7JX3; CR3022: PDB 7LOP; C118: PDB 7RKV. Identification of RBD residues within particular epitopes is approximate only, since assignments will vary depending upon which antibody is used for the representative member of each anti-RBD antibody class. **(B)** RBD amino acid sequence-based identity matrix based on the alignment in (A) for different sarbecovirus strains and SARS-2 VOCs.

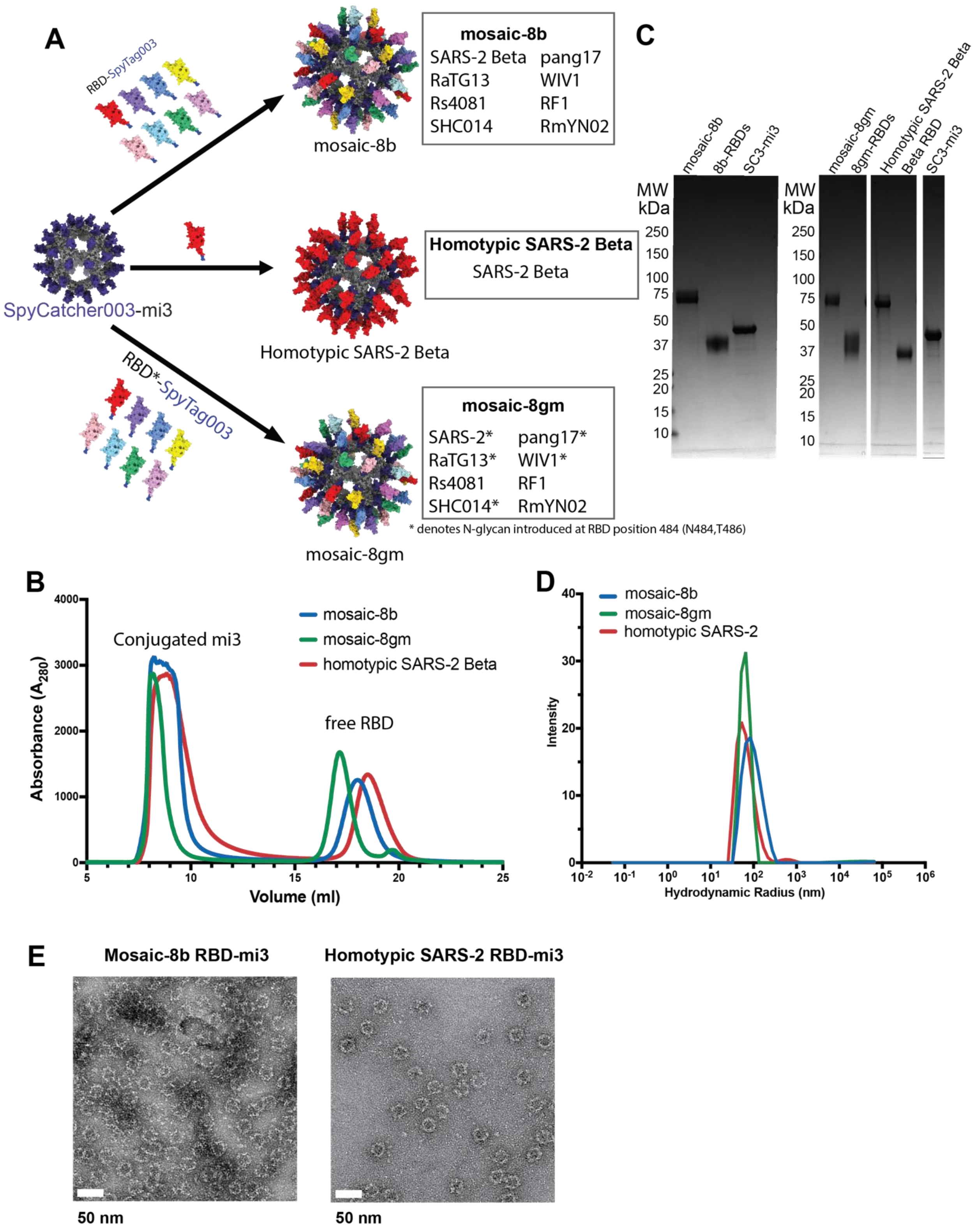
A
RBD-SpyTag003
SpyCatcher003-mi3
mosaic-8b
SARS-2 Beta
pang17
RaTG13
WIV1
Rs4081
RF1
SHC014
RmYN02
Homotypic SARS-2 Beta
SARS-2 Beta
RBD*-SpyTag003
mosaic-8gm
SARS-2*
pang17*
RaTG13*
WIV1*
Rs4081
RF1
SHC014*
RmYN02
* denotes N-glycan introduced at RBD position 484 (N484,T486)
C
mosaic-8b
8b-RBDs
SC3-mi3
mosaic-8gm
8gm-RBDs
Homotypic SARS-2 Beta
Beta RBD
SC3-mi3
MW kDa
250
150
100
75
50
37
25
20
15
10
B
Conjugated mi3
free RBD
mosaic-8b
mosaic-8gm
homotypic SARS-2 Beta
Absorbance (A280)
Volume (ml)
D
mosaic-8b
mosaic-8gm
homotypic SARS-2
Intensity
Hydrodynamic Radius (nm)
E
Mosaic-8b RBD-mi3
Homotypic SARS-2 RBD-mi3
50 nm
50 nm

Figure S2. Preparation of RBD-mi3 nanoparticles. (**A**) Schematic for construction of mosaic-8b, mosaic-8gm, and homotypic SARS-2 Beta RBD-mi3 nanoparticles. (**B**) Superose 6 10/300 SEC profile after RBD conjugations to mi3 (2-fold molar excess of RBD to mi3 subunit) showing peaks for RBD-mi3 nanoparticles and free RBD(s). (**C**) SDS-PAGE (Coomassie staining) of RBD-coupled nanoparticles, free RBDs, and free SpyCatcher003-mi3 particles (SC3-mi3). (**D**) Dynamic light scattering (DLS) measurements for RBD-coupled nanoparticles. Hydrodynamic radii were measured as 42 +/- 0.6 nm (n=10) (mosaic-8b), 33 +/- 0.9 nm (n=10) (mosaic-8gm), and 48 +/- 0.7 nm (n=10) (homotypic SARS-2). (**E**) Negative-stain EM images of RBD-coupled mi3 nanoparticles.

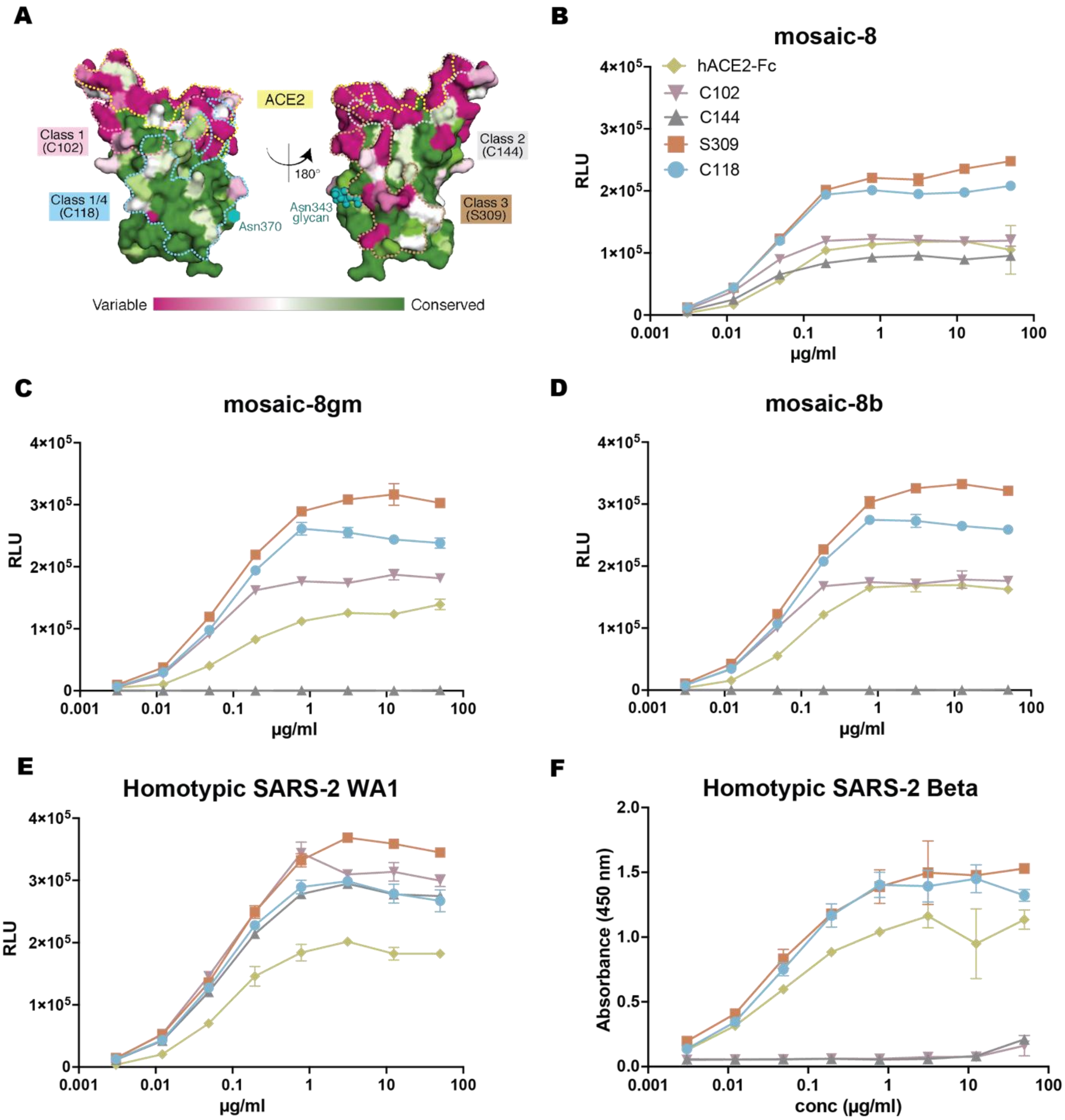
A
ACE2
Class 1
(C102)
Class 1/4
(C118)
Asn370
180°
Asn343
glycan
Class 2
(C144)
Class 3
(S309)
Variable
Conserved
B
mosaic-8
hACE2-Fc
C102
C144
S309
C118
RLU
µg/ml
C
mosaic-8gm
D
mosaic-8b
E
Homotypic SARS-2 WA1
F
Homotypic SARS-2 Beta
Absorbance (450 nm)
conc (µg/ml)

Figure S3. Binding characteristics of mosaic and homotypic RBD-mi3 nanoparticles. (**A**) Sequence conservation of the 16 sarbecovirus RBDs in Fig. 1D calculated by the ConSurf Database (*81*) shown on two views of an RBD surface (PDB 7BZ5). The ACE2 binding footprint (PDB 6M0J) is outlined by yellow dots. Epitopes of representative monoclonal antibodies used in binding experiments are outlined in dots of the indicated colors using information from structures of Fabs bound to RBD or S trimer (C118: PDB 7RKS, S309: PDB 7JX3; C144: PDB 7K90, C102: PDB 7K8M). The N-linked glycan attached to RBD residue 343 is indicated by teal spheres, and the potential N-linked glycosylation site at position 370 in RBDs derived from sarbecoviruses other than SARS-2 is indicated by a teal circle. (**B-F**) ELISAs to assess binding of the hACE2-Fc and the indicated monoclonal antibodies to RBD-mi3 nanoparticles. Nanoparticles were immobilized on an ELISA plate, incubated with the indicated monoclonal antibody or hACE2-Fc, and binding was detected using a labeled anti-human IgG secondary antibody. Data points are presented as the mean and standard deviation of duplicate measurements. Some error bars are too small to be distinguished from data points. RLU = relative luminescence units. (**B**) Binding to mosaic-8 RBD-mi3 (WA1 SARS-2 RBD plus seven animal sarbecovirus RBDs as previously described (*34*) and in fig. S2A). (**C**) Binding to mosaic-8gm RBD-mi3 (mosaic-8 with a WA1 SARS-2 RBD plus the seven animal sarbecovirus RBDs in which N-linked glycosylation site sequons at RBD position 484 were introduced in the clade 1a and 1b RBDs to occlude class 1 and 2 RBD epitopes). (**D**) Binding to mosaic-8b RBD-mi3 (SARS-2 Beta RBD plus the seven animal sarbecovirus RBDs in fig. S2A). (**E**) Binding to homotypic SARS-2 WA1 RBD-mi3 (as previously described (*34*)). (**F**) Binding to homotypic SARS-2 Beta RBD-mi3.

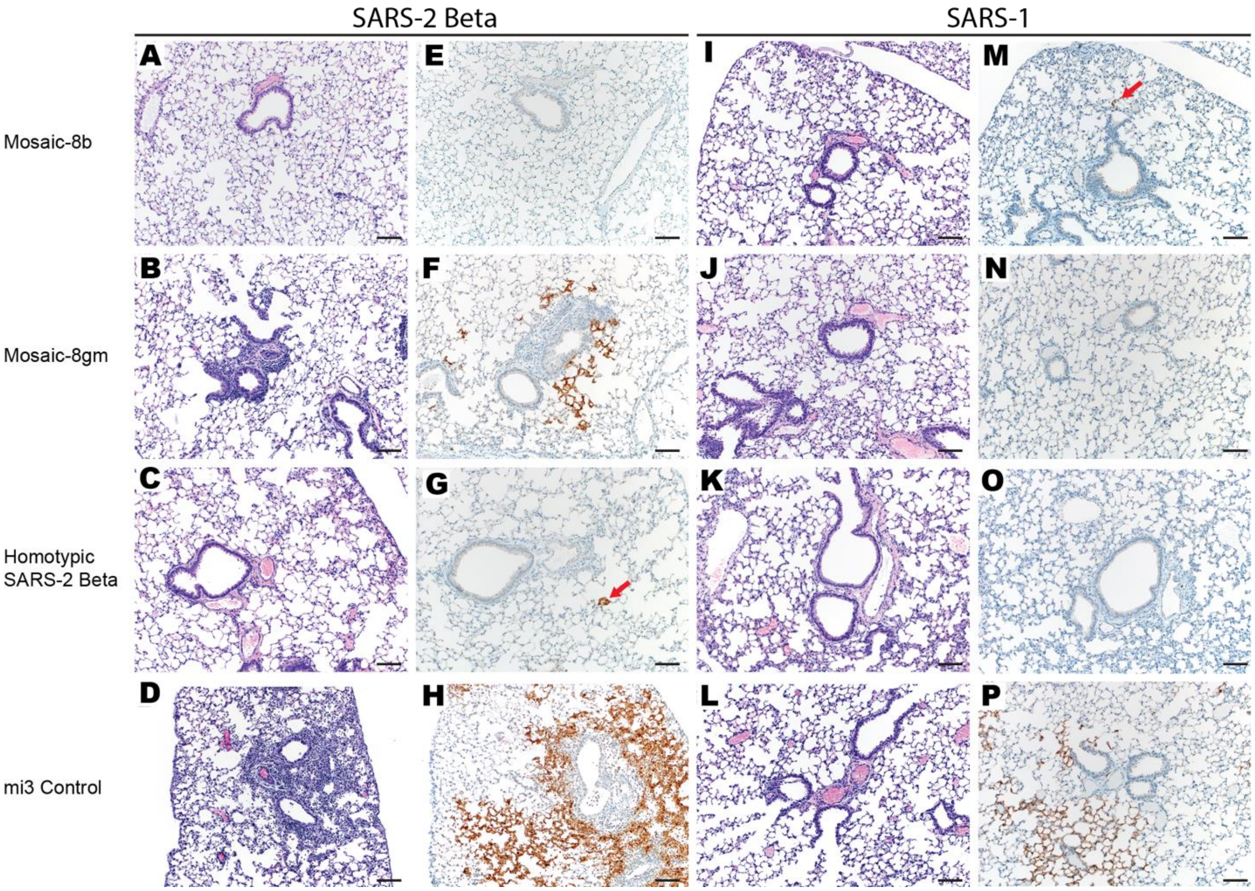

Q

		SARS-2 Beta															
	Lung	Mosiac-8b				Mosaic-8gm				Homotpyic SARS-2 Beta				mi3 control			
H & E Staining	Lesions %	0	1	0	0	0	0	5	20	0	0	0	0	<1	30	5	0
	Interstitial pneumonia	0	0	0	0	0	0	1	2	0	0	0	0	0	1	1	0
	Bronchiolitis	0	1	0	0	0	0	1	2	0	0	0	0	0	1	1	0
	perivascular leukocyte cuffing	0	0	0	0	0	0	1	2	0	0	0	1	1	3	1	0
IHC	Staining %	0	5	0	<1	0	<1	10	40	0	<1	<1	0	<1	90	20	0
	Type I and II pneumocytes	0	2	0	2	0	1	3	4	0	1	1	0	1	5	2	0

		SARS-1															
	Lung	Mosiac-8b				Mosaic-8gm				Homotpyic SARS-2 Beta				mi3 control			
H & E Staining	Lesions %	0	0	0	0	0	0	<1	0	0	0	0	0	0	5	0	0
	Interstitial pneumonia	0	0	0	0	0	0	1	0	0	0	0	0	0	1	0	0
	Bronchiolitis	0	0	0	0	0	0	1	0	0	0	0	0	0	1	0	0
	perivascular leukocyte cuffing	0	0	0	0	0	0	0	1	0	0	1	0	0	0	1	0
IHC	Staining %	<1	<1	0	0	0	<1	0	0	0	0	<1	0	10	90	60	50
	Type I and II pneumocytes	2	1	0	0	0	2	0	0	0	0	2	0	2	5	4	4

Figure S4. Lung pathology is reduced in mosaic-8b immunized mice challenged with either SARS-2 or SARS-1. Images taken at 100x magnification. Scale bar = 100µm. Red arrows = immunoreactivity in panels G and M. (**A-D)** Hematoxylin and eosin (H&E) stained lung tissue sections from animals vaccinated with either mosaic-8b, mosaic-8gm, homotypic SARS-2 Beta, or unconjugated mi3 and challenged with SARS-2 Beta (minimal-mild peribronchial inflammation in panels B and D). **(E-H)** Immunohistochemistry (IHC) staining for SARS-CoV-2 N protein antigen from animals vaccinated with either mosaic-8b, mosaic-8gm, homotypic SARS-2 Beta, or mi3 and challenged with SARS-2 Beta. **(I-L)** H&E stained lung tissue sections from animals vaccinated with either mosaic-8b, mosaic-8gm, homotypic SARS-2 Beta, or mi3 and challenged with SARS-1. **(M-P)** IHC staining for SARS-CoV-2 N protein antigen from animals vaccinated with either mosaic-8b, mosaic-8gm, homotypic SARS-2 Beta, or mi3 and challenged with SARS-1. (**Q**) Pathology and IHC for lung tissue isolated from vaccinated K18-hACE2 mice challenged with either SARS-2 Beta or SARS-1. Scoring for hematoxylin and eosin (H&E) is as follows: 0 = not present; 1 = minimal, 1-10%; 2 = mild, 11-25%; 3 = moderate, 26-50%; 4 = marked, 51-75%; 5 = severe, 76-100%. Scoring for IHC is as follows: 0 = not present; 1 = rare/few; 2 = scattered; 3 = moderate; 4 = numerous; 5 = diffuse. Each column represents a single animal.

Analysis: Upon challenge with SARS-2 Beta, two of four mi3 control animals exhibited lesions characterized by minimal interstitial pneumonia centered on terminal bronchioles and extending into the adjacent alveoli with minimal to moderate perivascular leucocyte cuffing and alveolar exudate (panels B,D,Q (top)). IHC for SARS-2 N protein showed staining in cells from three of four animals (<1-90% of type I and II cells) (panels D,H,Q (top)). In contrast, minimal to no lesions were observed in mosaic-8b and homotypic SARS-2 Beta immunized animals (panels A,C,Q (top)). Two of four animals immunized with mosaic-8gm exhibited lesions characterized by mild interstitial pneumonia. IHC staining for SARS-2 N protein showed minimal viral antigen present in mosaic-8b (1-5% of cells) and homotypic SARS-2 Beta immunized animals (two of four with <1% of stained cells) (panels E,G,Q (top)), whereas some staining was found in animals in the mosaic-8gm (three of four had <1-40% of stained type I and II pneumocytes). For the SARS-1 challenge, one animal in the control group exhibited lesions affecting 5% of the lung characterized by a minimal interstitial pneumonia centered on terminal bronchioles with minimal alveolar exudate (panels L,Q, (bottom)), whereas all four animals exhibited antigen staining (10-90% staining of type I and II cells) (panel P,Q (bottom)). In contrast, animals immunized with mosaic-8b did not have any observable pulmonary pathology (panel I,Q (bottom)). One animal vaccinated with mosaic-8gm had minimal interstitial pneumonia with peribronchial inflammation affecting less than 1% of the lung (panel J,Q (bottom)). Animals vaccinated with homotypic SARS-2 Beta did not have pulmonary lesions, except for one animal with minimal perivascular cuffing (panel K, Q (bottom)). In addition, animals vaccinated with mosaic-8b, mosaic-8gm, and homotypic SARS-2 Beta showed minimal to no antigen staining (all <1% in type I and II pneumocytes) (panel M-O,Q (bottom)). Overall, mosaic-8b immunization was efficacious against both SARS-1 and SARS-2 challenge. Mosaic-8gm immunized animals showed low levels of viral antigen staining in lung tissue obtained from SARS-1 challenged animals, but not SARS-2 challenged animals, whereas homotypic SARS-2 Beta immunized animals showed control of SARS-2 Beta and SARS-1 in the lungs matching the suppression of viral load in lung tissue (Fig. 3C,D). Of note, viral control in lung tissue did not match severity of disease for vaccinated animals (Fig 3A,B), most likely due to the neurological basis of disease severity in this animal model (*50*).

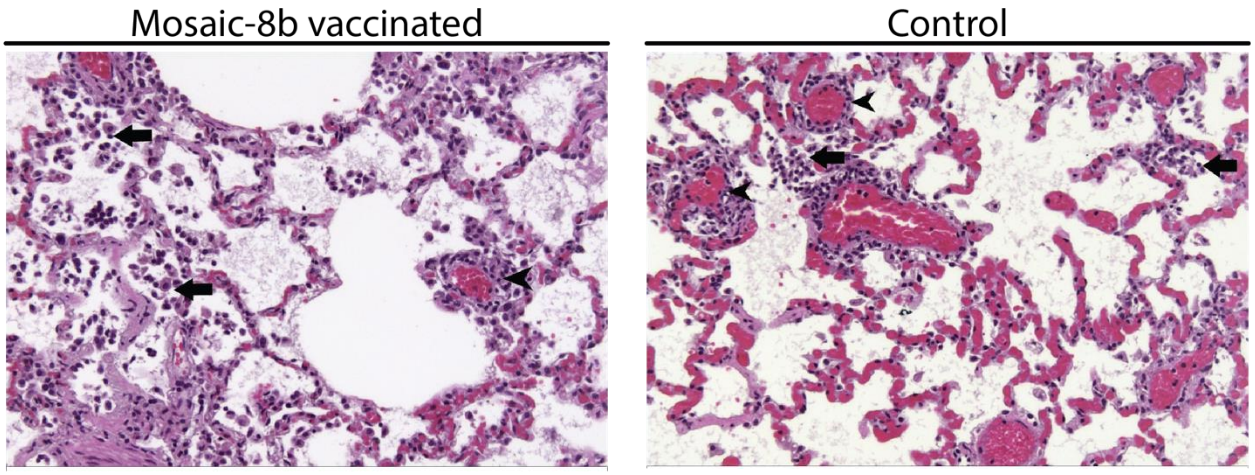

	Males		Females	
Groups	Mosaic-8b	control	Mosaic-8b	control
Animals/group	2	2	2	2
**LUNG**				
Inflammation, mixed or mononuclear, alveolar, bronchoalveolar and/or perivascular	2	2	1	1
minimal	1	2	1	1
mild	1	-	-	-
Alveolar macrophages, increased	2	1	2	0
minimal	2	1	2	-

Figure S5. Top: Lung pathology analyses in mosaic-8b and control immunized NHPs after SARS-2 Delta challenge revealed no detectable differences. Images were taken at 20x magnification. Left: representative image of lung tissue 4 days post challenge from mosaic-8b immunized animals. Right: representative image of lung tissue 4 days post challenge from control (adjuvant only) animals. Mononuclear cell inflammation was observed in alveolar spaces (arrows) and perivascularly (arrowheads). Bottom: Pathology for lung tissue isolated from vaccinated NHPs challenged with SARS-2 Delta. Histological score with % tissue involvement is as follows: *1 = minimal* <10%, *2 = mild*, 10-25%, *3 = moderate*, 25-40%, *4 = marked*, 50-95%, *5 = severe*, >95%.

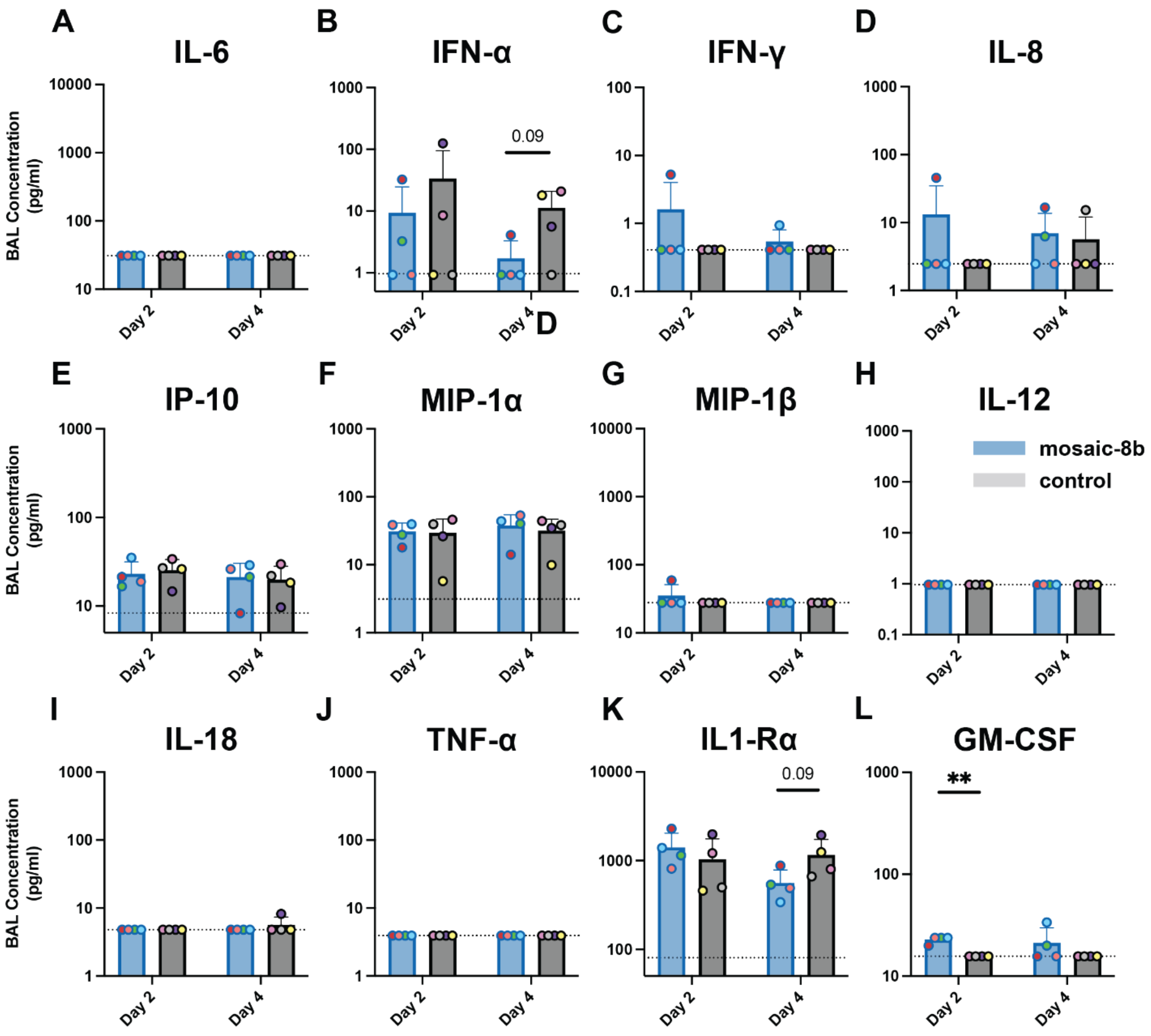

Figure S6. Cytokines and chemokines induced by SARS-2 Delta challenge in bronchial alveolar lavage (BAL) samples. Multiple cytokines and chemokines in Day 2 and Day 4 post challenge BAL samples were analyzed simultaneously using Luminex technology. Levels were measured for **(A)** IL-6, **(B)** IFN-α, **(C)** IFN-γ, **(D)** IL-8, **(E)** IP-10, **(F)** MIP-1α, **(G)** MIP-1β, **(H)** IL-12, **(I)** IL-18, **(J)** TNF-α, and **(K)** IL-1-Rα, **(L)** GM-CSF. Dashed horizontal lines correspond to background values representing the limit of detection. Significant differences between cohorts linked by horizontal lines are indicated by asterisks: $p<0.05$ = *, $p<0.01$ = **, $p<0.001$ = ***, $p<0.0001$ = ****

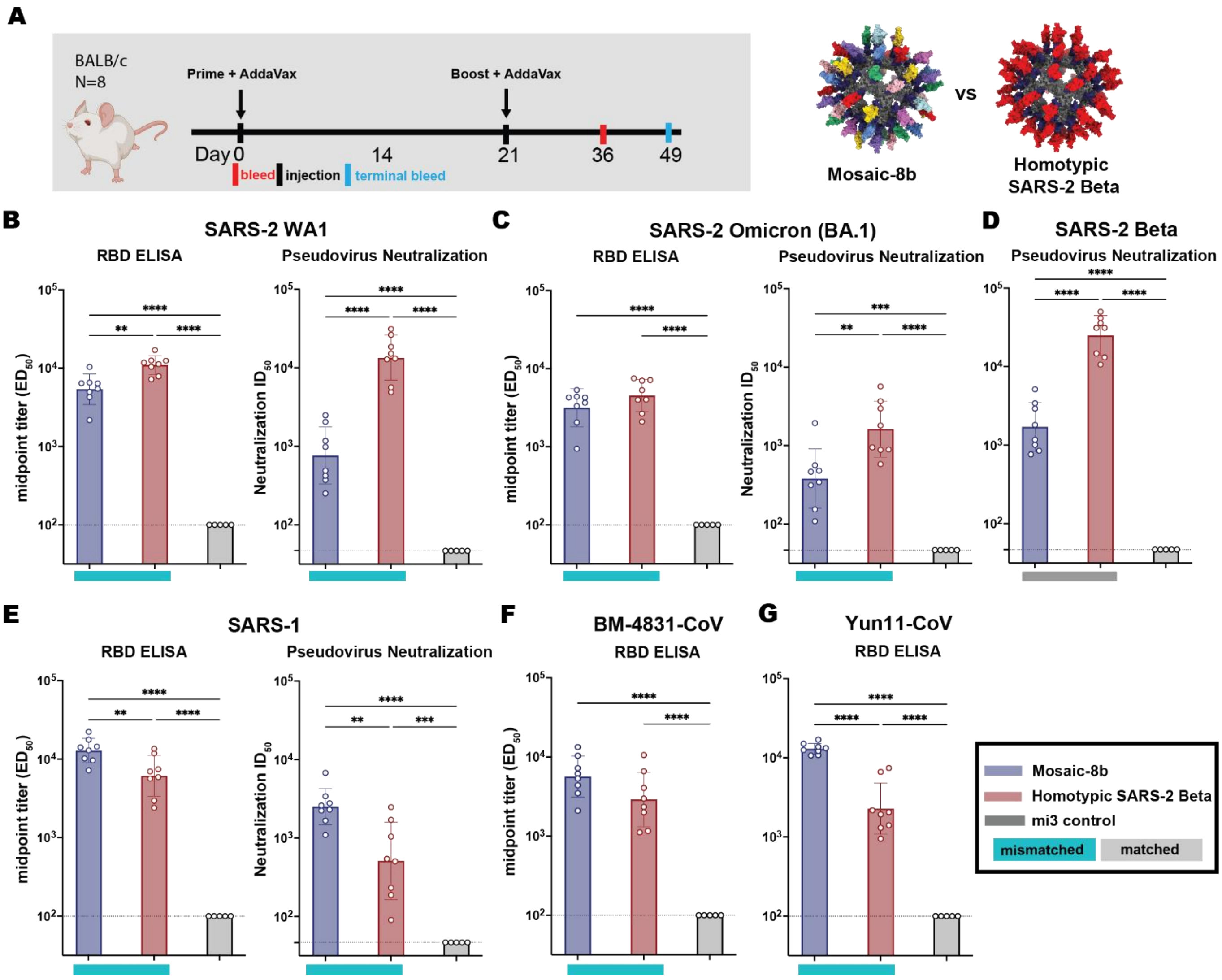

Figure S7. Mosaic-8b and homotypic SARS-2 Beta RBD-mi3 immunizations elicit binding and neutralizing antibodies in BALB/c mice. (**A**) Left: Immunization schedule. BALB/c mice were immunized with either mosaic-8 or homotypic SARS-2 Beta RBD-mi3. Right: Structural models of mosaic-8 and homotypic RBD-mi3 nanoparticles constructed using PDB 7SC1 (RBD), PDB 4MLI (SpyCatcher), and PDB 7B3Y (mi3). (**B-G**) ELISA and neutralization data for antisera (taken 4 weeks post boost) from individual mice (open circles) presented as the mean (bars) and standard deviation (horizontal lines). ELISA results are shown as midpoint titers (EC_{50} values); neutralization results are shown as half-maximal inhibitory dilutions (ID_{50} values). Dashed horizontal lines correspond to the background values representing the limit of detection. Significant differences between cohorts linked by horizontal lines are indicated by asterisks: $p<0.05$ = *, $p<0.01$ = **, $p<0.001$ = ***, $p<0.0001$ = ****. Rectangles below ELISA and neutralization data indicate mismatched strains (cyan; the RBD from that strain was not present on the nanoparticle) or matched strains (gray; the RBD was present on the nanoparticle).

A

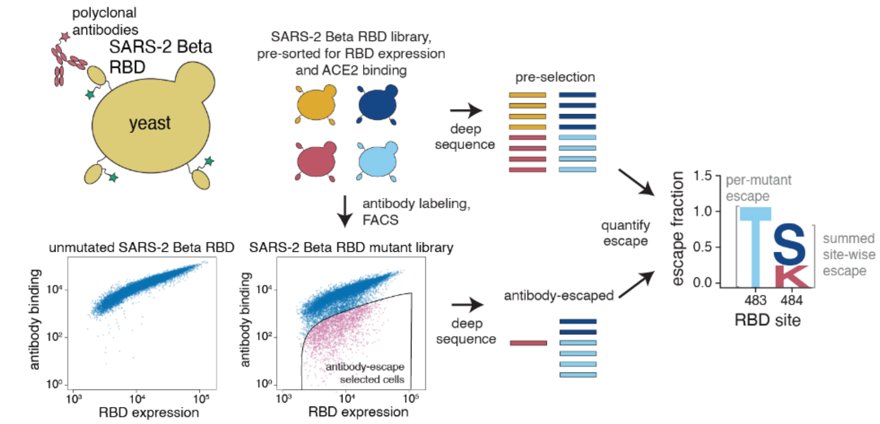

B

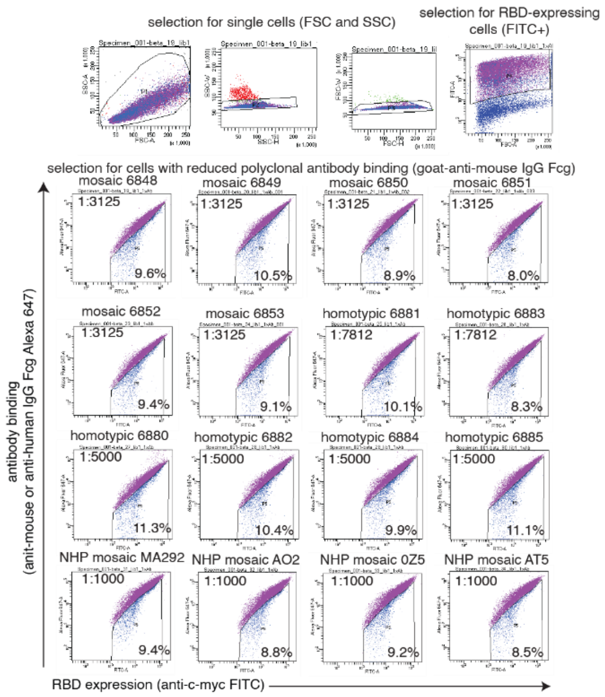

C

vaccine type	plasma	library	% cells in antibody-escape gate	estimated RBD+ cells processed on sorter
mosaic-8b RBD mouse sera	mosaic_6848	1	9.6	875492
		2	13.6	1002826
	mosaic_6849	1	10.5	957229
		2	13.2	1100621
	mosaic_6850	1	8.9	862307
		2	10.5	938801
	mosaic_6851	1	8	751160
		2	10.3	952949
	mosaic_6852	1	9.4	896191
		2	10.7	988504
	mosaic_6853	1	9.1	856793
		2	10.4	952068
homotypic RBD (Beta variant) mouse sera	homotypic_6881	1	10.1	973619
		2	8.6	826391
	homotypic_6883	1	8.3	787699
		2	8.5	853705
	homotypic_6880	1	11.3	8958239
		2	9	8536533
	homotypic_6882	1	10.4	7811471
		2	8.4	8672464
	homotypic_6884	1	9.9	8104919
		2	7.9	8084101
	homotypic_6885	1	11.1	8124820
		2	9	8108833
mosaic-8b RBD NHP sera	NHP_mosaic_MA292	1	9.4	11453946
		2	8.7	9252793
	NHP_mosaic_AO2	1	8.8	9921682
		2	8.6	8392547
	NHP_mosaic_0Z5	1	9.2	8802152
		2	8.3	9675928
	NHP_mosaic_AT5	1	8.5	9668012
		2	9.6	8889219

D

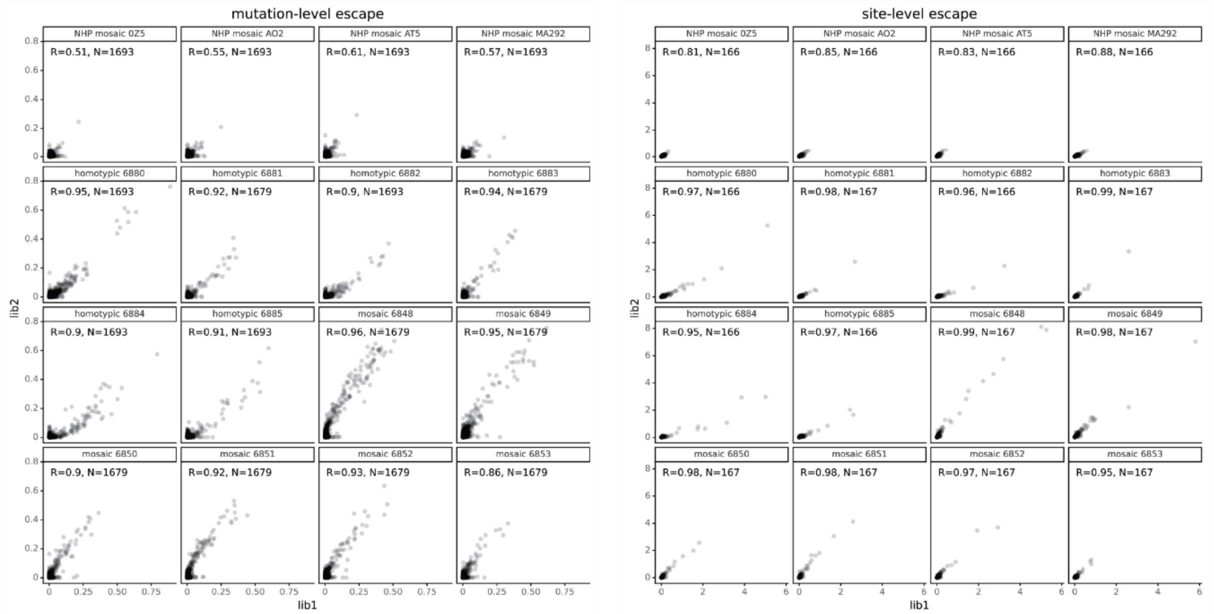

Figure S8. Comprehensive mapping of mutations that reduce binding of sera from immunized mice or NHPs to the SARS-2 Beta RBD. (**A**) Deep mutational scanning (*55*) was used to map mutations that reduced binding of polyclonal antibodies from immunized animals to the SARS-2 Beta RBD. A library of yeast containing nearly all possible mutations in the SARS-2 Beta RBD was incubated with sera from immunized mice or NHPs, and fluorescence-activated cell sorting (FACS) was used to enrich for cells expressing RBD (detected with a C-terminal Myc tag, green star) with reduced antibody binding, detected using an anti-mouse (for mouse sera) or anti-human (for NHP sera) IgG Fc-gamma secondary antibody. Deep sequencing was used to quantify the frequency of each mutation in the pre-selection and antibody-escape cell populations. We calculated each mutation's "escape fraction," the fraction of cells expressing RBD with that mutation that fell in the antibody-escape FACS bin (ranging from 0 to 1). The site-level escape metric is the sum of the escape fractions of all mutations at a site. (**B**) Top: Representative plots of nested FACS gating strategy used for all experiments to select for RBD+ single cells. Samples were gated by SSC-A versus FSC-A, SSC-W versus SSC-H, and FSC-W versus FSC-H) that also express RBD (FITC-A vs. FSC-A). Bottom: FACS gating strategy for one of two independent libraries to select cells expressing RBD mutants with reduced binding by polyclonal sera (cells in blue). Gates were set manually during sorting. Selection gates were set to capture cells that have a reduced amount of antibody binding for their degree of RBD expression. FACS scatter plots were qualitatively similar between the two libraries. SSC-A, side scatter-area; FSC-A, forward scatter-area; SSC-W, side scatter-width; SSC-H, side scatter-height; FSC-W, forward scatter-width; FSC-H, forward scatter height; FITC-A, fluorescein isothiocyanate-area. (**C**) The percent and number of RBD+ cells sorted into the antibody-escape gate for each library selected against each serum. (**D**) Mutation (top)- and site (bottom)-level correlations of escape scores between two independent biological replicate libraries. The complete antibody-escape scores are available in Data S3 and at https://github.com/jbloomlab/SARS-CoV-2-RBD_Beta_mosaic_np_vaccine/blob/main/results/supp_data/all_raw_data.csv.

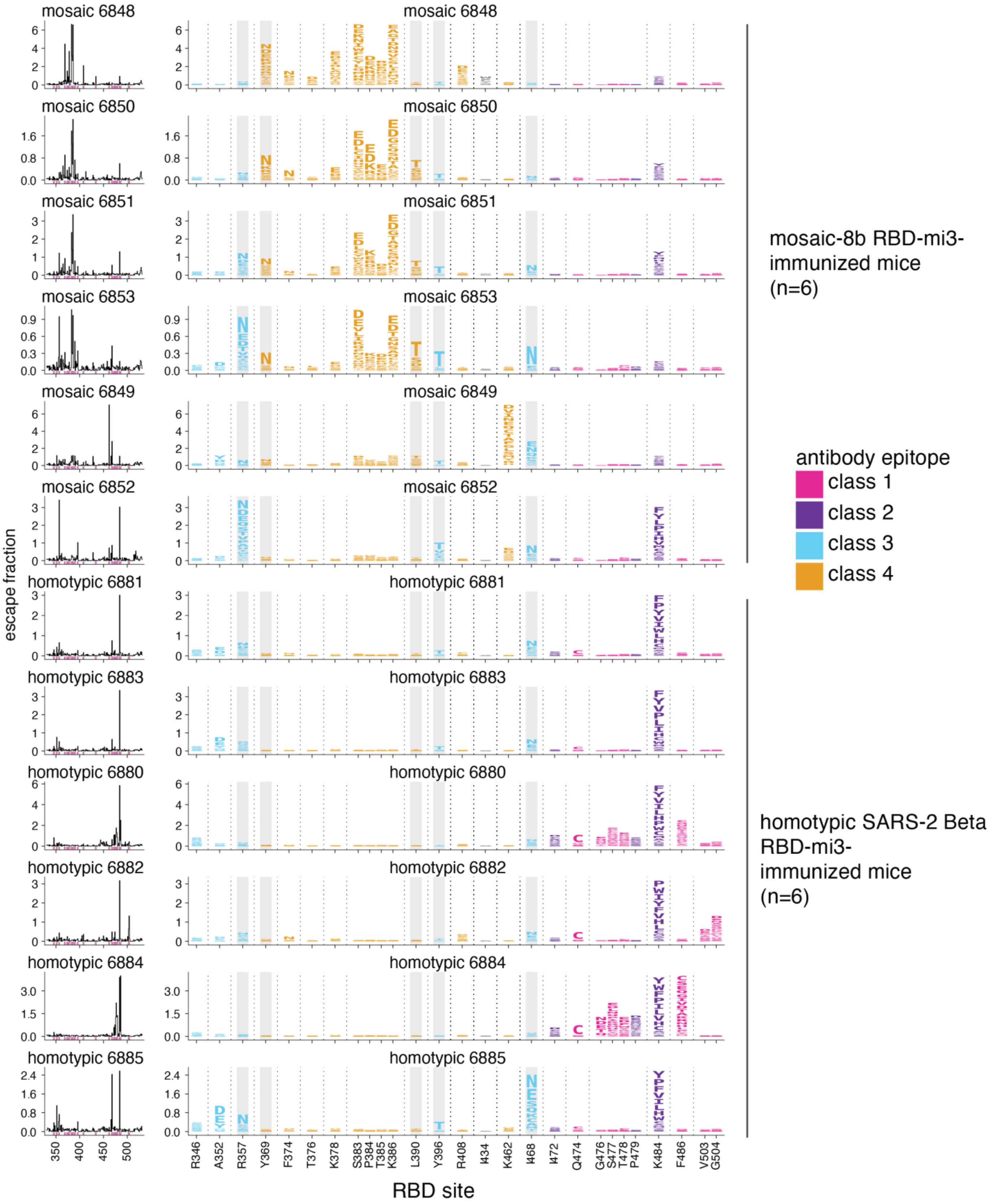

mosaic 6848
mosaic 6850
mosaic 6851
mosaic 6853
mosaic 6849
mosaic 6852
homotypic 6881
homotypic 6883
homotypic 6880
homotypic 6882
homotypic 6884
homotypic 6885
escape fraction
RBD site
R346 A352 R357 Y369 F374 T376 K378 S383 P384 T385 K386 L390 Y396 R408 I434 K462 I468 I472 Q474 G476 S477 T478 P479 K484 F486 V503 G504
350 400 450 500
mosaic-8b RBD-mi3-immunized mice (n=6)
antibody epitope
class 1
class 2
class 3
class 4
homotypic SARS-2 Beta RBD-mi3-immunized mice (n=6)

Figure S9. Complete antibody-escape maps for sera from mice immunized with the mosaic 8b-RBD-mi3 (top 6) or homotypic SARS-2 Beta RBD-mi3 (bottom 6) nanoparticles. The line plots at left indicate the sum of effects of all mutations at each RBD site on antibody binding, with larger values indicating more escape. The logo plots at right show key sites where mutations disrupted antibody binding (highlighted in purple on the line plot x-axes). The height of each letter is that mutation's escape fraction. The y-axis is scaled independently for each sample. RBD sites are colored by antibody epitope, indicated at right. Sites where some mutations introduce a potential N-linked glycosylation site sequon (NxS/T) are highlighted in gray. All escape scores are in Data S3 and at https://github.com/jbloomlab/SARS-CoV-2-RBD_Beta_mosaic_np_vaccine/blob/main/results/supp_data/all_raw_data.csv. Interactive versions of logo plots and structural visualizations are at https://jbloomlab.github.io/SARS-CoV-2-RBD_Beta_mosaic_np_vaccine/.

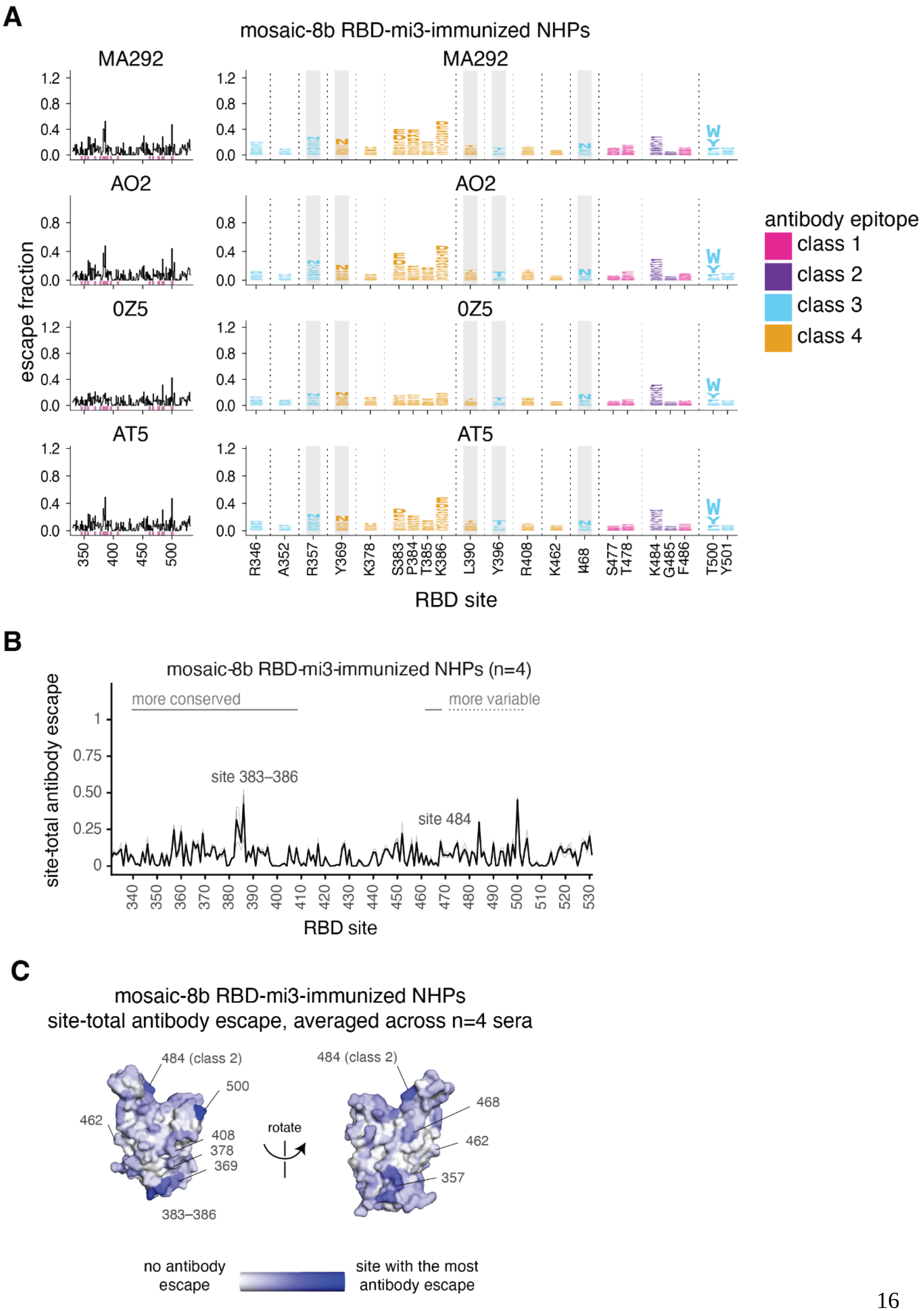
A
mosaic-8b RBD-mi3-immunized NHPs
MA292
AO2
0Z5
AT5
escape fraction
R346
A352
R357
Y369
K378
S383
P384
T385
K386
L390
Y396
R408
K462
I468
S477
T478
K484
G485
F486
T500
Y501
RBD site
antibody epitope
class 1
class 2
class 3
class 4
B
mosaic-8b RBD-mi3-immunized NHPs (n=4)
more conserved
more variable
site 383–386
site 484
site-total antibody escape
RBD site
C
mosaic-8b RBD-mi3-immunized NHPs
site-total antibody escape, averaged across n=4 sera
484 (class 2)
500
462
408
378
369
383–386
rotate
468
357
no antibody escape
site with the most antibody escape

Figure S10. Complete antibody-escape maps for sera from NHPs immunized with mosaic 8b-RBD-mi3. (**A**) As in fig. S9, line plots (left) and logo plots (right) indicate the sum of the escape fractions for each mutation at a site, or mutation-level escape fractions for key sites, respectively. The y-axis is scaled independently for each sample. Sites where mutations introduce a potential N-linked glycosylation site sequon (NxS/T) are highlighted in gray. RBD sites are colored by antibody epitope, indicated in panel B. **(B)** The site-total antibody escape is averaged across n=4 sera, with the y-axis scaled as in panel A. (C) The average site-total antibody escape is mapped to the surface of the SARS-2 Beta RBD (PDB 7LYQ), with white indicating no escape, and blue indicating the site with the most antibody escape. Key sites are labeled, and labels are colored by antibody class. All escape scores are in Data S3 and at https://github.com/jbloomlab/SARS-CoV-2-RBD_Beta_mosaic_np_vaccine/blob/main/results/supp_data/all_raw_data.csv. Interactive versions of logo plots and structural visualizations are at https://jbloomlab.github.io/SARS-CoV-2-RBD_Beta_mosaic_np_vaccine/.

References and Notes

1. C. Zheng, W. Shao, X. Chen, B. Zhang, G. Wang, W. Zhang, Real-world effectiveness of COVID-19 vaccines: A literature review and meta-analysis. *Int. J. Infect. Dis.* **114**, 252–260 (2022). doi:10.1016/j.ijid.2021.11.009 Medline

2. D. Planas, T. Bruel, L. Grzelak, F. Guivel-Benhassine, I. Staropoli, F. Porrot, C. Planchais, J. Buchrieser, M. M. Rajah, E. Bishop, M. Albert, F. Donati, M. Prot, S. Behillil, V. Enouf, M. Maquart, M. Smati-Lafarge, E. Varon, F. Schortgen, L. Yahyaoui, M. Gonzalez, J. De Sèze, H. Péré, D. Veyer, A. Sève, E. Simon-Lorière, S. Fafi-Kremer, K. Stefic, H. Mouquet, L. Hocqueloux, S. van der Werf, T. Prazuck, O. Schwartz, Sensitivity of infectious SARS-CoV-2 B.1.1.7 and B.1.351 variants to neutralizing antibodies. *Nat. Med.* **27**, 917–924 (2021). doi:10.1038/s41591-021-01318-5 Medline

3. N. L. Washington, K. Gangavarapu, M. Zeller, A. Bolze, E. T. Cirulli, K. M. Schiabor Barrett, B. B. Larsen, C. Anderson, S. White, T. Cassens, S. Jacobs, G. Levan, J. Nguyen, J. M. Ramirez 3rd, C. Rivera-Garcia, E. Sandoval, X. Wang, D. Wong, E. Spencer, R. Robles-Sikisaka, E. Kurzban, L. D. Hughes, X. Deng, C. Wang, V. Servellita, H. Valentine, P. De Hoff, P. Seaver, S. Sathe, K. Gietzen, B. Sickler, J. Antico, K. Hoon, J. Liu, A. Harding, O. Bakhtar, T. Basler, B. Austin, D. MacCannell, M. Isaksson, P. G. Febbo, D. Becker, M. Laurent, E. McDonald, G. W. Yeo, R. Knight, L. C. Laurent, E. de Feo, M. Worobey, C. Y. Chiu, M. A. Suchard, J. T. Lu, W. Lee, K. G. Andersen, Emergence and rapid transmission of SARS-CoV-2 B.1.1.7 in the United States. *Cell* **184**, 2587–2594.e7 (2021). doi:10.1016/j.cell.2021.03.052 Medline

4. T. K. Burki, Omicron variant and booster COVID-19 vaccines. *Lancet Respir. Med.* **10**, e17 (2022). doi:10.1016/S2213-2600(21)00559-2 Medline

5. L. Liu, S. Iketani, Y. Guo, J. F.-W. Chan, M. Wang, L. Liu, Y. Luo, H. Chu, Y. Huang, M. S. Nair, J. Yu, K. K.-H. Chik, T. T.-T. Yuen, C. Yoon, K. K.-W. To, H. Chen, M. T. Yin, M. E. Sobieszczyk, Y. Huang, H. H. Wang, Z. Sheng, K.-Y. Yuen, D. D. Ho, Striking antibody evasion manifested by the Omicron variant of SARS-CoV-2. *Nature* **602**, 676–681 (2022). doi:10.1038/s41586-021-04388-0 Medline

6. D. Yamasoba *et al.*, Virological characteristics of SARS-CoV-2 BA.2 variant. bioRxiv 480335 [Preprint] (2022); doi:10.1101/2022.02.14.480335.

7. F. Konings, M. D. Perkins, J. H. Kuhn, M. J. Pallen, E. J. Alm, B. N. Archer, A. Barakat, T. Bedford, J. N. Bhiman, L. Caly, L. L. Carter, A. Cullinane, T. de Oliveira, J. Druce, I. El Masry, R. Evans, G. F. Gao, A. E. Gorbalenya, E. Hamblion, B. L. Herring, E. Hodcroft, E. C. Holmes, M. Kakkar, S. Khare, M. P. G. Koopmans, B. Korber, J. Leite, D. MacCannell, M. Marklewitz, S. Maurer-Stroh, J. A. M. Rico, V. J. Munster, R. Neher, B. O. Munnink, B. I. Pavlin, M. Peiris, L. Poon, O. Pybus, A. Rambaut, P. Resende, L. Subissi, V. Thiel, S. Tong, S. van der Werf, A. von Gottberg, J. Ziebuhr, M. D. Van Kerkhove, SARS-CoV-2 Variants of Interest and Concern naming scheme conducive for global discourse. *Nat. Microbiol.* **6**, 821–823 (2021). doi:10.1038/s41564-021-00932-w Medline

8. S. Alkhovsky, S. Lenshin, A. Romashin, T. Vishnevskaya, O. Vyshemirsky, Y. Bulycheva, D. Lvov, A. Gitelman, SARS-like Coronaviruses in Horseshoe Bats (*Rhinolophus* spp.) in Russia, 2020. *Viruses* **14**, 113 (2022). doi:10.3390/v14010113 Medline

9. D. Delaune, V. Hul, E. A. Karlsson, A. Hassanin, T. P. Ou, A. Baidaliuk, F. Gámbaro, M. Prot, V. T. Tu, S. Chea, L. Keatts, J. Mazet, C. K. Johnson, P. Buchy, P. Dussart, T. Goldstein, E. Simon-Lorière, V. Duong, A novel SARS-CoV-2 related coronavirus in bats from Cambodia. *Nat. Commun.* **12**, 6563 (2021). doi:10.1038/s41467-021-26809-4 Medline

10. H. Zhou, J. Ji, X. Chen, Y. Bi, J. Li, Q. Wang, T. Hu, H. Song, R. Zhao, Y. Chen, M. Cui, Y. Zhang, A. C. Hughes, E. C. Holmes, W. Shi, Identification of novel bat coronaviruses sheds light on the evolutionary origins of SARS-CoV-2 and related viruses. *Cell* **184**, 4380–4391.e14 (2021). doi:10.1016/j.cell.2021.06.008 Medline

11. S. Wacharapluesadee, C. W. Tan, P. Maneeorn, P. Duengkae, F. Zhu, Y. Joyjinda, T. Kaewpom, W. N. Chia, W. Ampoot, B. L. Lim, K. Worachotsueptrakun, V. C.-W. Chen, N. Sirichan, C. Ruchisrisarod, A. Rodpan, K. Noradechanon, T. Phaichana, N. Jantarat, B. Thongnumchaima, C. Tu, G. Crameri, M. M. Stokes, T. Hemachudha, L.-F. Wang, Evidence for SARS-CoV-2 related coronaviruses circulating in bats and pangolins in Southeast Asia. *Nat. Commun.* **12**, 972 (2021). doi:10.1038/s41467-021-21240-1 Medline

12. S. N. Seifert, M. C. Letko, A sarbecovirus found in Russian bats uses human ACE2. bioRxiv 471310 [Preprint] (2021); doi:10.1101/2021.12.05.471310.

13. T. N. Starr, S. K. Zepeda, A. C. Walls, A. J. Greaney, S. Alkhovsky, D. Veesler, J. D. Bloom, ACE2 binding is an ancestral and evolvable trait of sarbecoviruses. *Nature* **603**, 913–918 (2022). doi:10.1038/s41586-022-04464-z Medline

14. M. Letko, A. Marzi, V. Munster, Functional assessment of cell entry and receptor usage for SARS-CoV-2 and other lineage B betacoronaviruses. *Nat. Microbiol.* **5**, 562–569 (2020). doi:10.1038/s41564-020-0688-y Medline

15. T. S. Fung, D. X. Liu, Human Coronavirus: Host-Pathogen Interaction. *Annu. Rev. Microbiol.* **73**, 529–557 (2019). doi:10.1146/annurev-micro-020518-115759 Medline

16. P. J. M. Brouwer, T. G. Caniels, K. van der Straten, J. L. Snitselaar, Y. Aldon, S. Bangaru, J. L. Torres, N. M. A. Okba, M. Claireaux, G. Kerster, A. E. H. Bentlage, M. M. van Haaren, D. Guerra, J. A. Burger, E. E. Schermer, K. D. Verheul, N. van der Velde, A. van der Kooi, J. van Schooten, M. J. van Breemen, T. P. L. Bijl, K. Sliepen, A. Aartse, R. Derking, I. Bontjer, N. A. Kootstra, W. J. Wiersinga, G. Vidarsson, B. L. Haagmans, A. B. Ward, G. J. de Bree, R. W. Sanders, M. J. van Gils, Potent neutralizing antibodies from COVID-19 patients define multiple targets of vulnerability. *Science* **369**, 643–650 (2020). doi:10.1126/science.abc5902 Medline

17. Y. Cao, B. Su, X. Guo, W. Sun, Y. Deng, L. Bao, Q. Zhu, X. Zhang, Y. Zheng, C. Geng, X. Chai, R. He, X. Li, Q. Lv, H. Zhu, W. Deng, Y. Xu, Y. Wang, L. Qiao, Y. Tan, L. Song, G. Wang, X. Du, N. Gao, J. Liu, J. Xiao, X. D. Su, Z. Du, Y. Feng, C. Qin, C. Qin, R. Jin, X. S. Xie, Potent neutralizing antibodies against SARS-CoV-2 identified by high-throughput single-cell sequencing of convalescent patients' B cells. *Cell* **182**, 73–84.e16 (2020). doi:10.1016/j.cell.2020.05.025 Medline

18. C. Kreer, M. Zehner, T. Weber, M. S. Ercanoglu, L. Gieselmann, C. Rohde, S. Halwe, M. Korenkov, P. Schommers, K. Vanshylla, V. Di Cristanziano, H. Janicki, R. Brinker, A. Ashurov, V. Krähling, A. Kupke, H. Cohen-Dvashi, M. Koch, J. M. Eckert, S. Lederer, N. Pfeifer, T. Wolf, M. J. G. T. Vehreschild, C. Wendtner, R. Diskin, H. Gruell, S.

Becker, F. Klein, Longitudinal Isolation of Potent Near-Germline SARS-CoV-2-Neutralizing Antibodies from COVID-19 Patients. *Cell* **182**, 843–854.e12 (2020). doi:10.1016/j.cell.2020.06.044 Medline

19. L. Liu, P. Wang, M. S. Nair, J. Yu, M. Rapp, Q. Wang, Y. Luo, J. F.-W. Chan, V. Sahi, A. Figueroa, X. V. Guo, G. Cerutti, J. Bimela, J. Gorman, T. Zhou, Z. Chen, K.-Y. Yuen, P. D. Kwong, J. G. Sodroski, M. T. Yin, Z. Sheng, Y. Huang, L. Shapiro, D. D. Ho, Potent neutralizing antibodies against multiple epitopes on SARS-CoV-2 spike. *Nature* **584**, 450–456 (2020). doi:10.1038/s41586-020-2571-7 Medline

20. D. F. Robbiani, C. Gaebler, F. Muecksch, J. C. C. Lorenzi, Z. Wang, A. Cho, M. Agudelo, C. O. Barnes, A. Gazumyan, S. Finkin, T. Hägglöf, T. Y. Oliveira, C. Viant, A. Hurley, H.-H. Hoffmann, K. G. Millard, R. G. Kost, M. Cipolla, K. Gordon, F. Bianchini, S. T. Chen, V. Ramos, R. Patel, J. Dizon, I. Shimeliovich, P. Mendoza, H. Hartweger, L. Nogueira, M. Pack, J. Horowitz, F. Schmidt, Y. Weisblum, E. Michailidis, A. W. Ashbrook, E. Waltari, J. E. Pak, K. E. Huey-Tubman, N. Koranda, P. R. Hoffman, A. P. West Jr., C. M. Rice, T. Hatziioannou, P. J. Bjorkman, P. D. Bieniasz, M. Caskey, M. C. Nussenzweig, Convergent antibody responses to SARS-CoV-2 in convalescent individuals. *Nature* **584**, 437–442 (2020). doi:10.1038/s41586-020-2456-9 Medline

21. R. Shi, C. Shan, X. Duan, Z. Chen, P. Liu, J. Song, T. Song, X. Bi, C. Han, L. Wu, G. Gao, X. Hu, Y. Zhang, Z. Tong, W. Huang, W. J. Liu, G. Wu, B. Zhang, L. Wang, J. Qi, H. Feng, F.-S. Wang, Q. Wang, G. F. Gao, Z. Yuan, J. Yan, A human neutralizing antibody targets the receptor-binding site of SARS-CoV-2. *Nature* **584**, 120–124 (2020). doi:10.1038/s41586-020-2381-y Medline

22. S. J. Zost, P. Gilchuk, R. E. Chen, J. B. Case, J. X. Reidy, A. Trivette, R. S. Nargi, R. E. Sutton, N. Suryadevara, E. C. Chen, E. Binshtein, S. Shrihari, M. Ostrowski, H. Y. Chu, J. E. Didier, K. W. MacRenaris, T. Jones, S. Day, L. Myers, F. Eun-Hyung Lee, D. C. Nguyen, I. Sanz, D. R. Martinez, P. W. Rothlauf, L.-M. Bloyet, S. P. J. Whelan, R. S. Baric, L. B. Thackray, M. S. Diamond, R. H. Carnahan, J. E. Crowe Jr., Rapid isolation and profiling of a diverse panel of human monoclonal antibodies targeting the SARS-CoV-2 spike protein. *Nat. Med.* **26**, 1422–1427 (2020). doi:10.1038/s41591-020-0998-x Medline

23. T. F. Rogers, F. Zhao, D. Huang, N. Beutler, A. Burns, W. T. He, O. Limbo, C. Smith, G. Song, J. Woehl, L. Yang, R. K. Abbott, S. Callaghan, E. Garcia, J. Hurtado, M. Parren, L. Peng, S. Ramirez, J. Ricketts, M. J. Ricciardi, S. A. Rawlings, N. C. Wu, M. Yuan, D. M. Smith, D. Nemazee, J. R. Teijaro, J. E. Voss, I. A. Wilson, R. Andrabi, B. Briney, E. Landais, D. Sok, J. G. Jardine, D. R. Burton, Isolation of potent SARS-CoV-2 neutralizing antibodies and protection from disease in a small animal model. *Science* **369**, 956–963 (2020). doi:10.1126/science.abc7520 Medline

24. E. Seydoux, L. J. Homad, A. J. MacCamy, K. R. Parks, N. K. Hurlburt, M. F. Jennewein, N. R. Akins, A. B. Stuart, Y.-H. Wan, J. Feng, R. E. Whaley, S. Singh, M. Boeckh, K. W. Cohen, M. J. McElrath, J. A. Englund, H. Y. Chu, M. Pancera, A. T. McGuire, L. Stamatatos, Analysis of a SARS-CoV-2-Infected Individual Reveals Development of Potent Neutralizing Antibodies with Limited Somatic Mutation. *Immunity* **53**, 98–105.e5 (2020). doi:10.1016/j.immuni.2020.06.001 Medline

25. S. J. Zost, P. Gilchuk, J. B. Case, E. Binshtein, R. E. Chen, J. P. Nkolola, A. Schäfer, J. X. Reidy, A. Trivette, R. S. Nargi, R. E. Sutton, N. Suryadevara, D. R. Martinez, L. E. Williamson, E. C. Chen, T. Jones, S. Day, L. Myers, A. O. Hassan, N. M. Kafai, E. S. Winkler, J. M. Fox, S. Shrihari, B. K. Mueller, J. Meiler, A. Chandrashekar, N. B. Mercado, J. J. Steinhardt, K. Ren, Y.-M. Loo, N. L. Kallewaard, B. T. McCune, S. P. Keeler, M. J. Holtzman, D. H. Barouch, L. E. Gralinski, R. S. Baric, L. B. Thackray, M. S. Diamond, R. H. Carnahan, J. E. Crowe Jr., Potently neutralizing and protective human antibodies against SARS-CoV-2. *Nature* **584**, 443–449 (2020). doi:10.1038/s41586-020-2548-6 Medline

26. C. O. Barnes, C. A. Jette, M. E. Abernathy, K. A. Dam, S. R. Esswein, H. B. Gristick, A. G. Malyutin, N. G. Sharaf, K. E. Huey-Tubman, Y. E. Lee, D. F. Robbiani, M. C. Nussenzweig, A. P. West Jr., P. J. Bjorkman, SARS-CoV-2 neutralizing antibody structures inform therapeutic strategies. *Nature* **588**, 682–687 (2020). doi:10.1038/s41586-020-2852-1 Medline

27. D. Pinto, Y.-J. Park, M. Beltramello, A. C. Walls, M. A. Tortorici, S. Bianchi, S. Jaconi, K. Culap, F. Zatta, A. De Marco, A. Peter, B. Guarino, R. Spreafico, E. Cameroni, J. B. Case, R. E. Chen, C. Havenar-Daughton, G. Snell, A. Telenti, H. W. Virgin, A. Lanzavecchia, M. S. Diamond, K. Fink, D. Veesler, D. Corti, Cross-neutralization of SARS-CoV-2 by a human monoclonal SARS-CoV antibody. *Nature* **583**, 290–295 (2020). doi:10.1038/s41586-020-2349-y Medline

28. L. Piccoli, Y.-J. Park, M. A. Tortorici, N. Czudnochowski, A. C. Walls, M. Beltramello, C. Silacci-Fregni, D. Pinto, L. E. Rosen, J. E. Bowen, O. J. Acton, S. Jaconi, B. Guarino, A. Minola, F. Zatta, N. Sprugasci, J. Bassi, A. Peter, A. De Marco, J. C. Nix, F. Mele, S. Jovic, B. F. Rodriguez, S. V. Gupta, F. Jin, G. Piumatti, G. Lo Presti, A. F. Pellanda, M. Biggiogero, M. Tarkowski, M. S. Pizzuto, E. Cameroni, C. Havenar-Daughton, M. Smithey, D. Hong, V. Lepori, E. Albanese, A. Ceschi, E. Bernasconi, L. Elzi, P. Ferrari, C. Garzoni, A. Riva, G. Snell, F. Sallusto, K. Fink, H. W. Virgin, A. Lanzavecchia, D. Corti, D. Veesler, Mapping neutralizing and immunodominant sites on the SARS-CoV-2 spike receptor-binding domain by structure-guided high-resolution serology. *Cell* **183**, 1024–1042.e21 (2020). doi:10.1016/j.cell.2020.09.037 Medline

29. Z. Wang, F. Schmidt, Y. Weisblum, F. Muecksch, C. O. Barnes, S. Finkin, D. Schaefer-Babajew, M. Cipolla, C. Gaebler, J. A. Lieberman, T. Y. Oliveira, Z. Yang, M. E. Abernathy, K. E. Huey-Tubman, A. Hurley, M. Turroja, K. A. West, K. Gordon, K. G. Millard, V. Ramos, J. Da Silva, J. Xu, R. A. Colbert, R. Patel, J. Dizon, C. Unson-O'Brien, I. Shimeliovich, A. Gazumyan, M. Caskey, P. J. Bjorkman, R. Casellas, T. Hatziioannou, P. D. Bieniasz, M. C. Nussenzweig, mRNA vaccine-elicited antibodies to SARS-CoV-2 and circulating variants. *Nature* **592**, 616–622 (2021). doi:10.1038/s41586-021-03324-6 Medline

30. H. Kleanthous, J. M. Silverman, K. W. Makar, I.-K. Yoon, N. Jackson, D. W. Vaughn, Scientific rationale for developing potent RBD-based vaccines targeting COVID-19. *NPJ Vaccines* **6**, 128 (2021). doi:10.1038/s41541-021-00393-6 Medline

31. H. Liu, N. C. Wu, M. Yuan, S. Bangaru, J. L. Torres, T. G. Caniels, J. van Schooten, X. Zhu, C. D. Lee, P. J. M. Brouwer, M. J. van Gils, R. W. Sanders, A. B. Ward, I. A. Wilson, Cross-Neutralization of a SARS-CoV-2 Antibody to a Functionally Conserved Site Is

Mediated by Avidity. *Immunity* **53**, 1272–1280.e5 (2020). doi:10.1016/j.immuni.2020.10.023 Medline

32. C. A. Jette, A. A. Cohen, P. N. P. Gnanapragasam, F. Muecksch, Y. E. Lee, K. E. Huey-Tubman, F. Schmidt, T. Hatziioannou, P. D. Bieniasz, M. C. Nussenzweig, A. P. West Jr., J. R. Keeffe, P. J. Bjorkman, C. O. Barnes, Broad cross-reactivity across sarbecoviruses exhibited by a subset of COVID-19 donor-derived neutralizing antibodies. *Cell Rep.* **36**, 109760 (2021). doi:10.1016/j.celrep.2021.109760 Medline

33. D. L. Burnett, K. J. L. Jackson, D. B. Langley, A. Aggrawal, A. O. Stella, M. D. Johansen, H. Balachandran, H. Lenthall, R. Rouet, G. Walker, B. M. Saunders, M. Singh, H. Li, J. Y. Henry, J. Jackson, A. G. Stewart, F. Witthauer, M. A. Spence, N. G. Hansbro, C. Jackson, P. Schofield, C. Milthorpe, M. Martinello, S. R. Schulz, E. Roth, A. Kelleher, S. Emery, W. J. Britton, W. D. Rawlinson, R. Karl, S. Schäfer, T. H. Winkler, R. Brink, R. A. Bull, P. M. Hansbro, H.-M. Jäck, S. Turville, D. Christ, C. C. Goodnow, Immunizations with diverse sarbecovirus receptor-binding domains elicit SARS-CoV-2 neutralizing antibodies against a conserved site of vulnerability. *Immunity* **54**, 2908–2921.e6 (2021). doi:10.1016/j.immuni.2021.10.019 Medline

34. A. A. Cohen, P. N. P. Gnanapragasam, Y. E. Lee, P. R. Hoffman, S. Ou, L. M. Kakutani, J. R. Keeffe, H.-J. Wu, M. Howarth, A. P. West, C. O. Barnes, M. C. Nussenzweig, P. J. Bjorkman, Mosaic nanoparticles elicit cross-reactive immune responses to zoonotic coronaviruses in mice. *Science* **371**, 735–741 (2021). doi:10.1126/science.abf6840 Medline

35. L. Bongini, D. Fanelli, F. Piazza, P. De Los Rios, M. Sanner, U. Skoglund, A dynamical study of antibody-antigen encounter reactions. *Phys. Biol.* **4**, 172–180 (2007). doi:10.1088/1478-3975/4/3/004 Medline

36. T. K. Tan, P. Rijal, R. Rahikainen, A. H. Keeble, L. Schimanski, S. Hussain, R. Harvey, J. W. P. Hayes, J. C. Edwards, R. K. McLean, V. Martini, M. Pedrera, N. Thakur, C. Conceicao, I. Dietrich, H. Shelton, A. Ludi, G. Wilsden, C. Browning, A. K. Zagrajek, D. Bialy, S. Bhat, P. Stevenson-Leggett, P. Hollinghurst, M. Tully, K. Moffat, C. Chiu, R. Waters, A. Gray, M. Azhar, V. Mioulet, J. Newman, A. S. Asfor, A. Burman, S. Crossley, J. A. Hammond, E. Tchilian, B. Charleston, D. Bailey, T. J. Tuthill, S. P. Graham, H. M. E. Duyvesteyn, T. Malinauskas, J. Huo, J. A. Tree, K. R. Buttigieg, R. J. Owens, M. W. Carroll, R. S. Daniels, J. W. McCauley, D. I. Stuart, K. A. Huang, M. Howarth, A. R. Townsend, A COVID-19 vaccine candidate using SpyCatcher multimerization of the SARS-CoV-2 spike protein receptor-binding domain induces potent neutralising antibody responses. *Nat. Commun.* **12**, 542 (2021). doi:10.1038/s41467-020-20654-7 Medline

37. K. D. Brune, D. B. Leneghan, I. J. Brian, A. S. Ishizuka, M. F. Bachmann, S. J. Draper, S. Biswas, M. Howarth, Plug-and-Display: Decoration of Virus-Like Particles via isopeptide bonds for modular immunization. *Sci. Rep.* **6**, 19234 (2016). doi:10.1038/srep19234 Medline

38. B. Zakeri, J. O. Fierer, E. Celik, E. C. Chittock, U. Schwarz-Linek, V. T. Moy, M. Howarth, Peptide tag forming a rapid covalent bond to a protein, through engineering a bacterial

adhesin. *Proc. Natl. Acad. Sci. U.S.A.* **109**, E690–E697 (2012). doi:10.1073/pnas.1115485109 Medline

39. A. H. Keeble, P. Turkki, S. Stokes, I. N. A. Khairil Anuar, R. Rahikainen, V. P. Hytönen, M. Howarth, Approaching infinite affinity through engineering of peptide-protein interaction. *Proc. Natl. Acad. Sci. U.S.A.* **116**, 26523–26533 (2019). doi:10.1073/pnas.1909653116 Medline

40. A. M. Davidson, J. Wysocki, D. Batlle, Interaction of SARS-CoV-2 and Other Coronavirus With ACE (Angiotensin-Converting Enzyme)-2 as Their Main Receptor: Therapeutic Implications. *Hypertension* **76**, 1339–1349 (2020). doi:10.1161/HYPERTENSIONAHA.120.15256 Medline

41. W. Dong, H. Mead, L. Tian, J.-G. Park, J. I. Garcia, S. Jaramillo, T. Barr, D. S. Kollath, V. K. Coyne, N. E. Stone, A. Jones, J. Zhang, A. Li, L.-S. Wang, M. Milanes-Yearsley, J. B. Torrelles, L. Martinez-Sobrido, P. S. Keim, B. M. Barker, M. A. Caligiuri, J. Yu, The K18-Human ACE2 Transgenic Mouse Model Recapitulates Non-severe and Severe COVID-19 in Response to an Infectious Dose of the SARS-CoV-2 Virus. *J. Virol.* **96**, e0096421 (2022). doi:10.1128/JVI.00964-21 Medline

42. C. K. Yinda, J. R. Port, T. Bushmaker, I. Offei Owusu, J. N. Purushotham, V. A. Avanzato, R. J. Fischer, J. E. Schulz, M. G. Holbrook, M. J. Hebner, R. Rosenke, T. Thomas, A. Marzi, S. M. Best, E. de Wit, C. Shaia, N. van Doremalen, V. J. Munster, K18-hACE2 mice develop respiratory disease resembling severe COVID-19. *PLOS Pathog.* **17**, e1009195 (2021). doi:10.1371/journal.ppat.1009195 Medline

43. E. S. Winkler, A. L. Bailey, N. M. Kafai, S. Nair, B. T. McCune, J. Yu, J. M. Fox, R. E. Chen, J. T. Earnest, S. P. Keeler, J. H. Ritter, L.-I. Kang, S. Dort, A. Robichaud, R. Head, M. J. Holtzman, M. S. Diamond, SARS-CoV-2 infection of human ACE2-transgenic mice causes severe lung inflammation and impaired function. *Nat. Immunol.* **21**, 1327–1335 (2020). doi:10.1038/s41590-020-0778-2 Medline

44. C. L. Hsieh, J. A. Goldsmith, J. M. Schaub, A. M. DiVenere, H.-C. Kuo, K. Javanmardi, K. C. Le, D. Wrapp, A. G. Lee, Y. Liu, C.-W. Chou, P. O. Byrne, C. K. Hjorth, N. V. Johnson, J. Ludes-Meyers, A. W. Nguyen, J. Park, N. Wang, D. Amengor, J. J. Lavinder, G. C. Ippolito, J. A. Maynard, I. J. Finkelstein, J. S. McLellan, Structure-based design of prefusion-stabilized SARS-CoV-2 spikes. *Science* **369**, 1501–1505 (2020). doi:10.1126/science.abd0826 Medline

45. A. J. Greaney, A. N. Loes, K. H. D. Crawford, T. N. Starr, K. D. Malone, H. Y. Chu, J. D. Bloom, Comprehensive mapping of mutations in the SARS-CoV-2 receptor-binding domain that affect recognition by polyclonal human plasma antibodies. *Cell Host Microbe* **29**, 463–476.e6 (2021). doi:10.1016/j.chom.2021.02.003 Medline

46. T. Zohar, C. Loos, S. Fischinger, C. Atyeo, C. Wang, M. D. Slein, J. Burke, J. Yu, J. Feldman, B. M. Hauser, T. Caradonna, A. G. Schmidt, Y. Cai, H. Streeck, E. T. Ryan, D. H. Barouch, R. C. Charles, D. A. Lauffenburger, G. Alter, Compromised humoral functional evolution tracks with SARS-CoV-2 mortality. *Cell* **183**, 1508–1519.e12 (2020). doi:10.1016/j.cell.2020.10.052 Medline

47. J. Yu, L. H. Tostanoski, L. Peter, N. B. Mercado, K. McMahan, S. H. Mahrokhian, J. P. Nkolola, J. Liu, Z. Li, A. Chandrashekar, D. R. Martinez, C. Loos, C. Atyeo, S.

Fischinger, J. S. Burke, M. D. Slein, Y. Chen, A. Zuiani, F. J. N. Lelis, M. Travers, S. Habibi, L. Pessaint, A. Van Ry, K. Blade, R. Brown, A. Cook, B. Finneyfrock, A. Dodson, E. Teow, J. Velasco, R. Zahn, F. Wegmann, E. A. Bondzie, G. Dagotto, M. S. Gebre, X. He, C. Jacob-Dolan, M. Kirilova, N. Kordana, Z. Lin, L. F. Maxfield, F. Nampanya, R. Nityanandam, J. D. Ventura, H. Wan, Y. Cai, B. Chen, A. G. Schmidt, D. R. Wesemann, R. S. Baric, G. Alter, H. Andersen, M. G. Lewis, D. H. Barouch, DNA vaccine protection against SARS-CoV-2 in rhesus macaques. *Science* **369**, 806–811 (2020). doi:10.1126/science.abc6284 Medline

48. N. van Doremalen, J. N. Purushotham, J. E. Schulz, M. G. Holbrook, T. Bushmaker, A. Carmody, J. R. Port, C. K. Yinda, A. Okumura, G. Saturday, F. Amanat, F. Krammer, P. W. Hanley, B. J. Smith, J. Lovaglio, S. L. Anzick, K. Barbian, C. Martens, S. C. Gilbert, T. Lambe, V. J. Munster, Intranasal ChAdOx1 nCoV-19/AZD1222 vaccination reduces viral shedding after SARS-CoV-2 D614G challenge in preclinical models. *Sci. Transl. Med.* **13**, eabh0755 (2021). doi:10.1126/scitranslmed.abh0755 Medline

49. G. Dagotto, N. B. Mercado, D. R. Martinez, Y. J. Hou, J. P. Nkolola, R. H. Carnahan, J. E. Crowe Jr., R. S. Baric, D. H. Barouch, Comparison of Subgenomic and Total RNA in SARS-CoV-2 Challenged Rhesus Macaques. *J. Virol.* **95**, e02370-20 (2021). doi:10.1128/JVI.02370-20 Medline

50. P. Kumari, H. A. Rothan, J. P. Natekar, S. Stone, H. Pathak, P. G. Strate, K. Arora, M. A. Brinton, M. Kumar, Neuroinvasion and Encephalitis Following Intranasal Inoculation of SARS-CoV-2 in K18-hACE2 Mice. *Viruses* **13**, 132 (2021). doi:10.3390/v13010132 Medline

51. B. Pulendran, P. S Arunachalam, D. T. O'Hagan, Emerging concepts in the science of vaccine adjuvants. *Nat. Rev. Drug Discov.* **20**, 454–475 (2021). doi:10.1038/s41573-021-00163-y Medline

52. P. B. Gilbert, D. C. Montefiori, A. B. McDermott, Y. Fong, D. Benkeser, W. Deng, H. Zhou, C. R. Houchens, K. Martins, L. Jayashankar, F. Castellino, B. Flach, B. C. Lin, S. O'Connell, C. McDanal, A. Eaton, M. Sarzotti-Kelsoe, Y. Lu, C. Yu, B. Borate, L. W. P. van der Laan, N. S. Hejazi, C. Huynh, J. Miller, H. M. El Sahly, L. R. Baden, M. Baron, L. De La Cruz, C. Gay, S. Kalams, C. F. Kelley, M. P. Andrasik, J. G. Kublin, L. Corey, K. M. Neuzil, L. N. Carpp, R. Pajon, D. Follmann, R. O. Donis, R. A. Koup; Immune Assays Team§; Moderna, Inc. Team§; Coronavirus Vaccine Prevention Network (CoVPN)/Coronavirus Efficacy (COVE) Team§; United States Government (USG)/CoVPN Biostatistics Team§, Immune correlates analysis of the mRNA-1273 COVID-19 vaccine efficacy clinical trial. *Science* **375**, 43–50 (2022). Medline

53. F. Hansen, K. Meade-White, C. Clancy, R. Rosenke, A. Okumura, D. W. Hawman, F. Feldmann, B. Kaza, M. A. Jarvis, K. Rosenke, H. Feldmann, SARS-CoV-2 reinfection prevents acute respiratory disease in Syrian hamsters but not replication in the upper respiratory tract. *Cell Rep.* **38**, 110515 (2022). doi:10.1016/j.celrep.2022.110515 Medline

54. A. J. Greaney, T. N. Starr, P. Gilchuk, S. J. Zost, E. Binshtein, A. N. Loes, S. K. Hilton, J. Huddleston, R. Eguia, K. H. D. Crawford, A. S. Dingens, R. S. Nargi, R. E. Sutton, N. Suryadevara, P. W. Rothlauf, Z. Liu, S. P. J. Whelan, R. H. Carnahan, J. E. Crowe Jr., J. D. Bloom, Complete Mapping of Mutations to the SARS-CoV-2 Spike Receptor-Binding

Domain that Escape Antibody Recognition. *Cell Host Microbe* **29**, 44–57.e9 (2021). doi:10.1016/j.chom.2020.11.007 Medline

55. A. J. Greaney, T. N. Starr, R. T. Eguia, A. N. Loes, K. Khan, F. Karim, S. Cele, J. E. Bowen, J. K. Logue, D. Corti, D. Veesler, H. Y. Chu, A. Sigal, J. D. Bloom, A SARS-CoV-2 variant elicits an antibody response with a shifted immunodominance hierarchy. *PLOS Pathog.* **18**, e1010248 (2022). doi:10.1371/journal.ppat.1010248 Medline

56. A. C. Walls *et al*., Distinct sensitivities to SARS-CoV-2 variants in vaccinated humans and mice. bioRxiv 479468 [Preprint] (2022); doi:10.1101/2022.02.07.479468.

57. R. Shinnakasu, S. Sakakibara, H. Yamamoto, P. H. Wang, S. Moriyama, N. Sax, C. Ono, A. Yamanaka, Y. Adachi, T. Onodera, T. Sato, M. Shinkai, R. Suzuki, Y. Matsuura, N. Hashii, Y. Takahashi, T. Inoue, K. Yamashita, T. Kurosaki, Glycan engineering of the SARS-CoV-2 receptor-binding domain elicits cross-neutralizing antibodies for SARS-related viruses. *J. Exp. Med.* **218**, e20211003 (2021). doi:10.1084/jem.20211003 Medline

58. J. Heeney *et al*., Gene delivery of a single,structurally engineered Coronavirus vaccine antigen elicits SARS-CoV-2 Omicron and pan-Sarbecovirus neutralisation. *Research Square* 995273 [Preprint] (2021); doi:10.21203/rs.3.rs-995273/v1.

59. W. Dejnirattisai, D. Zhou, H. M. Ginn, H. M. E. Duyvesteyn, P. Supasa, J. B. Case, Y. Zhao, T. S. Walter, A. J. Mentzer, C. Liu, B. Wang, G. C. Paesen, J. Slon-Campos, C. López-Camacho, N. M. Kafai, A. L. Bailey, R. E. Chen, B. Ying, C. Thompson, J. Bolton, A. Fyfe, S. Gupta, T. K. Tan, J. Gilbert-Jaramillo, W. James, M. Knight, M. W. Carroll, D. Skelly, C. Dold, Y. Peng, R. Levin, T. Dong, A. J. Pollard, J. C. Knight, P. Klenerman, N. Temperton, D. R. Hall, M. A. Williams, N. G. Paterson, F. K. R. Bertram, C. A. Siebert, D. K. Clare, A. Howe, J. Radecke, Y. Song, A. R. Townsend, K. A. Huang, E. E. Fry, J. Mongkolsapaya, M. S. Diamond, J. Ren, D. I. Stuart, G. R. Screaton, The antigenic anatomy of SARS-CoV-2 receptor binding domain. *Cell* **184**, 2183–2200.e22 (2021). doi:10.1016/j.cell.2021.02.032 Medline

60. A. J. Greaney, T. N. Starr, C. O. Barnes, Y. Weisblum, F. Schmidt, M. Caskey, C. Gaebler, A. Cho, M. Agudelo, S. Finkin, Z. Wang, D. Poston, F. Muecksch, T. Hatziioannou, P. D. Bieniasz, D. F. Robbiani, M. C. Nussenzweig, P. J. Bjorkman, J. D. Bloom, Mapping mutations to the SARS-CoV-2 RBD that escape binding by different classes of antibodies. *Nat. Commun.* **12**, 4196 (2021). doi:10.1038/s41467-021-24435-8 Medline

61. T. N. Starr, N. Czudnochowski, Z. Liu, F. Zatta, Y.-J. Park, A. Addetia, D. Pinto, M. Beltramello, P. Hernandez, A. J. Greaney, R. Marzi, W. G. Glass, I. Zhang, A. S. Dingens, J. E. Bowen, M. A. Tortorici, A. C. Walls, J. A. Wojcechowskyj, A. De Marco, L. E. Rosen, J. Zhou, M. Montiel-Ruiz, H. Kaiser, J. R. Dillen, H. Tucker, J. Bassi, C. Silacci-Fregni, M. P. Housley, J. di Iulio, G. Lombardo, M. Agostini, N. Sprugasci, K. Culap, S. Jaconi, M. Meury, E. Dellota Jr., R. Abdelnabi, S. C. Foo, E. Cameroni, S. Stumpf, T. I. Croll, J. C. Nix, C. Havenar-Daughton, L. Piccoli, F. Benigni, J. Neyts, A. Telenti, F. A. Lempp, M. S. Pizzuto, J. D. Chodera, C. M. Hebner, H. W. Virgin, S. P. J. Whelan, D. Veesler, D. Corti, J. D. Bloom, G. Snell, SARS-CoV-2 RBD antibodies that maximize breadth and resistance to escape. *Nature* **597**, 97–102 (2021). doi:10.1038/s41586-021-03807-6 Medline

62. T. N. Starr, A. J. Greaney, A. Addetia, W. W. Hannon, M. C. Choudhary, A. S. Dingens, J. Z. Li, J. D. Bloom, Prospective mapping of viral mutations that escape antibodies used to treat COVID-19. *Science* **371**, 850–854 (2021). doi:10.1126/science.abf9302 Medline

63. E. Cameroni, J. E. Bowen, L. E. Rosen, C. Saliba, S. K. Zepeda, K. Culap, D. Pinto, L. A. VanBlargan, A. De Marco, J. di Iulio, F. Zatta, H. Kaiser, J. Noack, N. Farhat, N. Czudnochowski, C. Havenar-Daughton, K. R. Sprouse, J. R. Dillen, A. E. Powell, A. Chen, C. Maher, L. Yin, D. Sun, L. Soriaga, J. Bassi, C. Silacci-Fregni, C. Gustafsson, N. M. Franko, J. Logue, N. T. Iqbal, I. Mazzitelli, J. Geffner, R. Grifantini, H. Chu, A. Gori, A. Riva, O. Giannini, A. Ceschi, P. Ferrari, P. E. Cippà, A. Franzetti-Pellanda, C. Garzoni, P. J. Halfmann, Y. Kawaoka, C. Hebner, L. A. Purcell, L. Piccoli, M. S. Pizzuto, A. C. Walls, M. S. Diamond, A. Telenti, H. W. Virgin, A. Lanzavecchia, G. Snell, D. Veesler, D. Corti, Broadly neutralizing antibodies overcome SARS-CoV-2 Omicron antigenic shift. *Nature* **602**, 664–670 (2022). doi:10.1038/s41586-021-04386-2 Medline

64. D. J. Sheward *et al*., Structural basis of Omicron neutralization by affinity-matured public antibodies. bioRxiv 474825 [Preprint] (2022); doi:10.1101/2022.01.03.474825.

65. K. Westendorf, S. Žentelis, L. Wang, D. Foster, P. Vaillancourt, M. Wiggin, E. Lovett, R. van der Lee, J. Hendle, A. Pustilnik, J. M. Sauder, L. Kraft, Y. Hwang, R. W. Siegel, J. Chen, B. A. Heinz, R. E. Higgs, N. L. Kallewaard, K. Jepson, R. Goya, M. A. Smith, D. W. Collins, D. Pellacani, P. Xiang, V. de Puyraimond, M. Ricicova, L. Devorkin, C. Pritchard, A. O'Neill, K. Dalal, P. Panwar, H. Dhupar, F. A. Garces, C. A. Cohen, J. M. Dye, K. E. Huie, C. V. Badger, D. Kobasa, J. Audet, J. J. Freitas, S. Hassanali, I. Hughes, L. Munoz, H. C. Palma, B. Ramamurthy, R. W. Cross, T. W. Geisbert, V. Menachery, K. Lokugamage, V. Borisevich, I. Lanz, L. Anderson, P. Sipahimalani, K. S. Corbett, E. S. Yang, Y. Zhang, W. Shi, T. Zhou, M. Choe, J. Misasi, P. D. Kwong, N. J. Sullivan, B. S. Graham, T. L. Fernandez, C. L. Hansen, E. Falconer, J. R. Mascola, B. E. Jones, B. C. Barnhart, LY-CoV1404 (bebtelovimab) potently neutralizes SARS-CoV-2 variants. *Cell Rep.* **39**, 110812 (2022). doi:10.1016/j.celrep.2022.110812 Medline

66. M. G. Joyce, W.-H. Chen, R. S. Sankhala, A. Hajduczki, P. V. Thomas, M. Choe, E. J. Martinez, W. C. Chang, C. E. Peterson, E. B. Morrison, C. Smith, R. E. Chen, A. Ahmed, L. Wieczorek, A. Anderson, J. B. Case, Y. Li, T. Oertel, L. Rosado, A. Ganesh, C. Whalen, J. M. Carmen, L. Mendez-Rivera, C. P. Karch, N. Gohain, Z. Villar, D. McCurdy, Z. Beck, J. Kim, S. Shrivastava, O. Jobe, V. Dussupt, S. Molnar, U. Tran, C. B. Kannadka, S. Soman, C. Kuklis, M. Zemil, H. Khanh, W. Wu, M. A. Cole, D. K. Duso, L. W. Kummer, T. J. Lang, S. E. Muncil, J. R. Currier, S. J. Krebs, V. R. Polonis, S. Rajan, P. M. McTamney, M. T. Esser, W. W. Reiley, M. Rolland, N. de Val, M. S. Diamond, G. D. Gromowski, G. R. Matyas, M. Rao, N. L. Michael, K. Modjarrad, SARS-CoV-2 ferritin nanoparticle vaccines elicit broad SARS coronavirus immunogenicity. *Cell Rep.* **37**, 110143 (2021). doi:10.1016/j.celrep.2021.110143 Medline

67. K. O. Saunders, E. Lee, R. Parks, D. R. Martinez, D. Li, H. Chen, R. J. Edwards, S. Gobeil, M. Barr, K. Mansouri, S. M. Alam, L. L. Sutherland, F. Cai, A. M. Sanzone, M. Berry, K. Manne, K. W. Bock, M. Minai, B. M. Nagata, A. B. Kapingidza, M. Azoitei, L. V. Tse, T. D. Scobey, R. L. Spreng, R. W. Rountree, C. T. DeMarco, T. N. Denny, C. W.

Woods, E. W. Petzold, J. Tang, T. H. Oguin 3rd, G. D. Sempowski, M. Gagne, D. C. Douek, M. A. Tomai, C. B. Fox, R. Seder, K. Wiehe, D. Weissman, N. Pardi, H. Golding, S. Khurana, P. Acharya, H. Andersen, M. G. Lewis, I. N. Moore, D. C. Montefiori, R. S. Baric, B. F. Haynes, Neutralizing antibody vaccine for pandemic and pre-emergent coronaviruses. *Nature* **594**, 553–559 (2021). doi:10.1038/s41586-021-03594-0 Medline

68. A. E. Powell, K. Zhang, M. Sanyal, S. Tang, P. A. Weidenbacher, S. Li, T. D. Pham, J. E. Pak, W. Chiu, P. S. Kim, A Single Immunization with Spike-Functionalized Ferritin Vaccines Elicits Neutralizing Antibody Responses against SARS-CoV-2 in Mice. *ACS Cent. Sci.* **7**, 183–199 (2021). doi:10.1021/acscentsci.0c01405 Medline

69. P. T. Heath, E. P. Galiza, D. N. Baxter, M. Boffito, D. Browne, F. Burns, D. R. Chadwick, R. Clark, C. Cosgrove, J. Galloway, A. L. Goodman, A. Heer, A. Higham, S. Iyengar, A. Jamal, C. Jeanes, P. A. Kalra, C. Kyriakidou, D. F. McAuley, A. Meyrick, A. M. Minassian, J. Minton, P. Moore, I. Munsoor, H. Nicholls, O. Osanlou, J. Packham, C. H. Pretswell, A. San Francisco Ramos, D. Saralaya, R. P. Sheridan, R. Smith, R. L. Soiza, P. A. Swift, E. C. Thomson, J. Turner, M. E. Viljoen, G. Albert, I. Cho, F. Dubovsky, G. Glenn, J. Rivers, A. Robertson, K. Smith, S. Toback; 2019nCoV-302 Study Group, Safety and Efficacy of NVX-CoV2373 Covid-19 Vaccine. *N. Engl. J. Med.* **385**, 1172–1183 (2021). doi:10.1056/NEJMoa2107659 Medline

70. W. Wang, B. Huang, Y. Zhu, W. Tan, M. Zhu, Ferritin nanoparticle-based SARS-CoV-2 RBD vaccine induces a persistent antibody response and long-term memory in mice. *Cell. Mol. Immunol.* **18**, 749–751 (2021). doi:10.1038/s41423-021-00643-6 Medline

71. X. Ma, F. Zou, F. Yu, R. Li, Y. Yuan, Y. Zhang, X. Zhang, J. Deng, T. Chen, Z. Song, Y. Qiao, Y. Zhan, J. Liu, J. Zhang, X. Zhang, Z. Peng, Y. Li, Y. Lin, L. Liang, G. Wang, Y. Chen, Q. Chen, T. Pan, X. He, H. Zhang, Nanoparticle Vaccines Based on the Receptor Binding Domain (RBD) and Heptad Repeat (HR) of SARS-CoV-2 Elicit Robust Protective Immune Responses. *Immunity* **53**, 1315–1330.e9 (2020). doi:10.1016/j.immuni.2020.11.015 Medline

72. Q. Geng, W. Tai, V. K. Baxter, J. Shi, Y. Wan, X. Zhang, S. A. Montgomery, S. A. Taft-Benz, E. J. Anderson, A. C. Knight, K. H. Dinnon 3rd, S. R. Leist, R. S. Baric, J. Shang, S.-W. Hong, A. Drelich, C. K. Tseng, M. Jenkins, M. Heise, L. Du, F. Li, Novel virus-like nanoparticle vaccine effectively protects animal model from SARS-CoV-2 infection. *PLOS Pathog.* **17**, e1009897 (2021). doi:10.1371/journal.ppat.1009897 Medline

73. Y. F. Kang, C. Sun, Z. Zhuang, R.-Y. Yuan, Q. Zheng, J.-P. Li, P.-P. Zhou, X.-C. Chen, Z. Liu, X. Zhang, X.-H. Yu, X.-W. Kong, Q.-Y. Zhu, Q. Zhong, M. Xu, N.-S. Zhong, Y.-X. Zeng, G.-K. Feng, C. Ke, J.-C. Zhao, M.-S. Zeng, Rapid Development of SARS-CoV-2 Spike Protein Receptor-Binding Domain Self-Assembled Nanoparticle Vaccine Candidates. *ACS Nano* **15**, 2738–2752 (2021). doi:10.1021/acsnano.0c08379 Medline

74. A. C. Walls, B. Fiala, A. Schäfer, S. Wrenn, M. N. Pham, M. Murphy, L. V. Tse, L. Shehata, M. A. O'Connor, C. Chen, M. J. Navarro, M. C. Miranda, D. Pettie, R. Ravichandran, J. C. Kraft, C. Ogohara, A. Palser, S. Chalk, E.-C. Lee, K. Guerriero, E. Kepl, C. M. Chow, C. Sydeman, E. A. Hodge, B. Brown, J. T. Fuller, K. H. Dinnon 3rd, L. E. Gralinski, S. R. Leist, K. L. Gully, T. B. Lewis, M. Guttman, H. Y. Chu, K. K. Lee, D. H. Fuller, R. S.

Baric, P. Kellam, L. Carter, M. Pepper, T. P. Sheahan, D. Veesler, N. P. King, Elicitation of Potent Neutralizing Antibody Responses by Designed Protein Nanoparticle Vaccines for SARS-CoV-2. *Cell* **183**, 1367–1382.e17 (2020). doi:10.1016/j.cell.2020.10.043 Medline

75. D. Li *et al.*, Breadth of SARS-CoV-2 Neutralization and Protection Induced by a Nanoparticle Vaccine. bioRxiv 477915 [Preprint] (2022); doi:10.1101/2022.01.26.477915.

76. A. I. Mosa, Antigenic Variability. *Front. Immunol.* **11**, 2057 (2020). doi:10.3389/fimmu.2020.02057 Medline

77. M. Kanekiyo, M. G. Joyce, R. A. Gillespie, J. R. Gallagher, S. F. Andrews, H. M. Yassine, A. K. Wheatley, B. E. Fisher, D. R. Ambrozak, A. Creanga, K. Leung, E. S. Yang, S. Boyoglu-Barnum, I. S. Georgiev, Y. Tsybovsky, M. S. Prabhakaran, H. Andersen, W.-P. Kong, U. Baxa, K. L. Zephir, J. E. Ledgerwood, R. A. Koup, P. D. Kwong, A. K. Harris, A. B. McDermott, J. R. Mascola, B. S. Graham, Mosaic nanoparticle display of diverse influenza virus hemagglutinins elicits broad B cell responses. *Nat. Immunol.* **20**, 362–372 (2019). doi:10.1038/s41590-018-0305-x Medline

78. A. C. Walls, M. C. Miranda, A. Schäfer, M. N. Pham, A. Greaney, P. S. Arunachalam, M.-J. Navarro, M. A. Tortorici, K. Rogers, M. A. O'Connor, L. Shirreff, D. E. Ferrell, J. Bowen, N. Brunette, E. Kepl, S. K. Zepeda, T. Starr, C.-L. Hsieh, B. Fiala, S. Wrenn, D. Pettie, C. Sydeman, K. R. Sprouse, M. Johnson, A. Blackstone, R. Ravichandran, C. Ogohara, L. Carter, S. W. Tilles, R. Rappuoli, S. R. Leist, D. R. Martinez, M. Clark, R. Tisch, D. T. O'Hagan, R. Van Der Most, W. C. Van Voorhis, D. Corti, J. S. McLellan, H. Kleanthous, T. P. Sheahan, K. D. Smith, D. H. Fuller, F. Villinger, J. Bloom, B. Pulendran, R. S. Baric, N. P. King, D. Veesler, Elicitation of broadly protective sarbecovirus immunity by receptor-binding domain nanoparticle vaccines. *Cell* **184**, 5432–5447.e16 (2021). doi:10.1016/j.cell.2021.09.015 Medline

79. C. Fan *et al.*, Neutralizing monoclonal antibodies elicited by mosaic RBD nanoparticles bind conserved sarbecovirus epitopes. bioRxiv 497989 [Preprint] (2022); doi:10.1101/2022.06.28.497989.

80. T. U. J. Bruun, A. C. Andersson, S. J. Draper, M. Howarth, Engineering a Rugged Nanoscaffold To Enhance Plug-and-Display Vaccination. *ACS Nano* **12**, 8855–8866 (2018). doi:10.1021/acsnano.8b02805 Medline

81. C. O. Barnes, A. P. West Jr., K. E. Huey-Tubman, M. A. G. Hoffmann, N. G. Sharaf, P. R. Hoffman, N. Koranda, H. B. Gristick, C. Gaebler, F. Muecksch, J. C. C. Lorenzi, S. Finkin, T. Hägglöf, A. Hurley, K. G. Millard, Y. Weisblum, F. Schmidt, T. Hatziioannou, P. D. Bieniasz, M. Caskey, D. F. Robbiani, M. C. Nussenzweig, P. J. Bjorkman, Structures of Human Antibodies Bound to SARS-CoV-2 Spike Reveal Common Epitopes and Recurrent Features of Antibodies. *Cell* **182**, 828–842.e16 (2020). doi:10.1016/j.cell.2020.06.025 Medline

82. A. A. Cohen, Z. Yang, P. N. P. Gnanapragasam, S. Ou, K. A. Dam, H. Wang, P. J. Bjorkman, Construction, characterization, and immunization of nanoparticles that display a diverse array of influenza HA trimers. *PLOS ONE* **16**, e0247963 (2021). doi:10.1371/journal.pone.0247963 Medline

83. L. J. Reed, H. Muench, A Simple Method of Estimating Fifty Per Cent Endpoints12. *Am. J. Epidemiol.* **27**, 493–497 (1938). doi:10.1093/oxfordjournals.aje.a118408

84. K. H. D. Crawford, R. Eguia, A. S. Dingens, A. N. Loes, K. D. Malone, C. R. Wolf, H. Y. Chu, M. A. Tortorici, D. Veesler, M. Murphy, D. Pettie, N. P. King, A. B. Balazs, J. D. Bloom, Protocol and Reagents for Pseudotyping Lentiviral Particles with SARS-CoV-2 Spike Protein for Neutralization Assays. *Viruses* **12**, 513 (2020). doi:10.3390/v12050513 Medline

85. S. N. Seifert *et al.*, An ACE2-dependent Sarbecovirus in Russian bats is resistant to SARS-CoV-2 vaccines. bioRxiv 471310 [Preprint] (2022); doi:10.1101/2021.12.05.471310.

86. A. P. West Jr., L. Scharf, J. Horwitz, F. Klein, M. C. Nussenzweig, P. J. Bjorkman, Computational analysis of anti-HIV-1 antibody neutralization panel data to identify potential functional epitope residues. *Proc. Natl. Acad. Sci. U.S.A.* **110**, 10598–10603 (2013). doi:10.1073/pnas.1309215110 Medline

87. T. N. Starr, A. J. Greaney, S. K. Hilton, D. Ellis, K. H. D. Crawford, A. S. Dingens, M. J. Navarro, J. E. Bowen, M. A. Tortorici, A. C. Walls, N. P. King, D. Veesler, J. D. Bloom, Deep Mutational Scanning of SARS-CoV-2 Receptor Binding Domain Reveals Constraints on Folding and ACE2 Binding. *Cell* **182**, 1295–1310.e20 (2020). doi:10.1016/j.cell.2020.08.012 Medline

88. S. K. Hilton, J. Huddleston, A. Black, K. North, A. S. Dingens, T. Bedford, J. D. Bloom, *dms-view*: Interactive visualization tool for deep mutational scanning data. *J. Open Source Softw.* **5**, 2353 (2020). doi:10.21105/joss.02353 Medline

89. M. Landau, I. Mayrose, Y. Rosenberg, F. Glaser, E. Martz, T. Pupko, N. Ben-Tal, ConSurf 2005: The projection of evolutionary conservation scores of residues on protein structures. *Nucleic Acids Res.* **33**, W299-302 (2005). doi:10.1093/nar/gki370 Medline

90. S. Guindon, J.-F. Dufayard, V. Lefort, M. Anisimova, W. Hordijk, O. Gascuel, New algorithms and methods to estimate maximum-likelihood phylogenies: Assessing the performance of PhyML 3.0. *Syst. Biol.* **59**, 307–321 (2010). doi:10.1093/sysbio/syq010 Medline

91. F. Sievers, A. Wilm, D. Dineen, T. J. Gibson, K. Karplus, W. Li, R. Lopez, H. McWilliam, M. Remmert, J. Söding, J. D. Thompson, D. G. Higgins, Fast, scalable generation of high-quality protein multiple sequence alignments using Clustal Omega. *Mol. Syst. Biol.* **7**, 539 (2011). doi:10.1038/msb.2011.75 Medline
