# Supplementary material for: Mosaic RBD nanoparticles protect against challenge by diverse sarbecoviruses in animal models
Source: Science. 2022 Jul 5:eabq0839. doi: 10.1126/science.abq0839 (PMC9273039; doi:10.1126/science.abq0839)
Supplement: Supplementary file 3 — Data S1 to S3 [file science.abq0839_sm_data_s1_to_s3.zip › science.abq0839_sm-data-s2.docx]

BIOQUAL, Inc.

STUDY NUMBER 100161.00.001

(PROTOCOL No. 21-092p)

EPL PROJECT NUMBER 391-128

EVALUATION IN NON-HUMAN PRIMATES OF PROTECTION FROM SARS-Cov-2 INFECTION AFTER IMMUNIZATION WITH MOSAIC NANOPARTICLE CANDIDATE VACCINE

1^ST^ DRAFT PATHOLOGY REPORT

Submitted by:

Experimental Pathology Laboratories, Inc.

Street Address: Mailing Address:

45600 Terminal Drive P.O. Box 169

Sterling, VA 20166 Sterling, VA 20167-0169

(703) 471-7060

Submitted to:

BIOQUAL, Inc.

Rockville, MD 20850-3336

April 29, 2022

**FINAL REPORT**

| TABLE OF CONTENTS | | |
| --- | --- | --- |
|  | Page | |
| PATHOLOGY NARRATIVE | | 1 |
| INCIDENCE SUMMARY OF MICROSCOPIC FINDINGS BY SACRIFICE | | 1 / 4 |
| INDIVIDUAL DATA LISTING OF HISTOPATHOLOGY | | 1 / 8 |
| SEVERITY GRADING SCALE | |  |
| APPENDIX A: FIGURES AND LEGENDS | | A-1 |

1^ST^ DRAFT PATHOLOGY NARRATIVE

BIOQUAL, Inc.

STUDY NUMBER 100161.00.001

(PROTOCOL No. 21-092p)

EPL PROJECT NUMBER 391-128

EVALUATION IN NON-HUMAN PRIMATES OF PROTECTION FROM SARS-CoV-2 INFECTION AFTER IMMUNIZATION WITH MOSAIC NANOPARTICLE CANDIDATE VACCINE

1^ST^ DRAFT PATHOLOGY REPORT

**OBJECTIVE**

The objective of this study was to determine the protection against SARS-CoV-2 viral disease after immunization with a mosaic nanoparticle candidate vaccine in non-human primates.

**MATERIALS AND METHODS**

A total of eight (8) non-human primates were on study. There were two groups with four animals/group (2 males / 2 females). Animals were naïve or received Mosaic-8 RBD and GMP VAC20 intramuscularly. Treated animals were administered vaccine on Study Days (SD) 0, 28, and 92. All animals were challenged with SARS-CoV-2 Delta intranasal/intratracheal on SD 119. Animals were euthanized on SD 123, four days after viral challenge, for tissue collection. See Study Design, Text Table 1.

Text Table 1. Study Design

| Group | N | Treatment (IM)  SD 0, 28 and 92 | Challenge  (IN/IT)  SD 119 | Tissue Collection  SD 123 |
| --- | --- | --- | --- | --- |
| 1 | 4 (2 males / 2 females) | Mosaic-8 RBD and GMP VAC20 | SARS-CoV-2 | Collect lung |
| 3 | 4 (2 males / 2 females) | Control |  |  |

At necropsy, lung was collected and placed in 10% neutral buffered formalin for histopathologic analysis. Tissue sections were trimmed and processed to hematoxylin and eosin (H&E) stained slides and examined by a board-certified pathologist at Experimental Pathology Laboratories, Inc. (EPL^®^) in Sterling, Virginia. Histopathologic findings are presented in the Individual Data Listing of Histopathology tables. Findings were graded from one to five, depending upon severity and summarized by treatment group in the Incidence Summary of Microscopic Findings by Sacrifice. An explanation of descriptive severity grades is provided at the end of the tables; equivalent numbered grades are 1 = minimal, 2 = mild, 3 = moderate, 4 = marked, 5 = severe.

**RESULTS**

**MORTALITY**

There were no found dead or early moribund sacrifices.

**MACROSCOPIC FINDINGS**

Macroscopic findings were not observed.

**MICROSCOPIC FINDINGS**

In all animals, treated and untreated, microscopic findings in the lung were minimal to mild.

In one Group 3 animal (Animal CN48), foreign bodies were multifocally associated with mixed or mononuclear cell inflammation. Foreign bodies included plant material and keratinized epithelium. Inhalation of plant, food or other small particles with associated inflammation can be observed as background lesions in non-human primates but may also have been introduced from intratracheal installations and/or bronchoalveolar lavage. These findings are unrelated to SARS-CoV-2 exposure and cannot be distinguished from SARS-CoV-2 microscopic findings; this animal was, therefore, excluded from comparison to other animals.

Bronchus-associated lymphoid tissue (BALT) is a normal anatomic feature of the lung but can vary in amount depending on immunologic responses to antigens. The presence and amount of BALT was quantified per section to assess differences between groups.

Alveolar macrophages are resident phagocytic cells that remove foreign agents in the lung, cell populations can fluctuate due to variable background changes. Their presence is considered normal but may increase with exposure to various agents.

Although lung sections were examined and scored separately for microscopic findings, the histologic score per lung section is equivalent to a severity identified for lung overall. For ease of visualization, findings were consolidated per animal with the highest severity identified in Text Table 2, below. For all microscopic findings by lung lobe, see Incidence Summary of Microscopic Findings by Sacrifice tables for incidence and severity of findings by group.

Text Table 2. Microscopic findings in the lung

|  | Males | | Females | |
| --- | --- | --- | --- | --- |
| Groups | 1 | 3 | 1 | 3 |
| Animals/group | 2 | 2 | 2 | 2 |
| **LUNG** |  |  |  |  |
| Inflammation, mixed or mononuclear, alveolar, bronchoalveolar and/or perivascular | 2 | 2 | 1 | 1 |
| minimal | 1 | 2 | 1 | 1 |
| mild | 1 | - | - | - |
| Alveolar macrophages, increased | 2 | 1 | 2 | 0 |
| minimal | 2 | 1 | 2 | - |

Representative images of groups can be reviewed in Appendix A: Figures and Legends.

**DISCUSSION**

There was no sufficient magnitude of difference in microscopic findings of the lung in SARS-CoV-2 only animals (Group 3) when compared to mosaic nanoparticle candidate vaccine treated animals (Group 1).

| SHANNON M. WALLACE, DVM, DABT, Diplomate, ACVP  Senior Pathologist |
| --- |

SMW/cb

INCIDENCE SUMMARY OF MICROSCOPIC FINDINGS BY SACRIFICE

INDIVIDUAL DATA LISTING OF HISTOPATHOLOGY

**SEVERITY GRADING SCALE**

The severity of the non-neoplastic tissue lesions are graded as follows:

**Grade 1 (1+): Minimal.** This corresponds to a histopathologic change ranging from inconspicuous to barely noticeable but so minor, small, or infrequent as to warrant no more than the least assignable grade. For multifocal or diffusely-distributed lesions, this grade was used for processes where less than approximately10% of the tissue in an average high-power field was involved. For focal or diffuse hyperplastic/hypoplastic/ atrophic lesions, this grade was used when the affected structure or tissue had undergone a less than approximately 10% increase or decrease in volume.

**Grade 2 (2+) Mild.** This corresponds to a histopathologic change that is a noticeable but not a prominent feature of the tissue. For multifocal or diffusely-distributed lesions, this grade was used for processes where between approximately 10% and 25% of the tissue in an average high-power field was involved. For focal or diffuse hyperplastic/hypoplastic/atrophic lesions, this grade was used when the affected structure or tissue had undergone between an approximately 10% to 25% increase or decrease in volume.

**Grade 3 (3+): Moderate.** This corresponds to a histopathologic change that is a prominent but not a dominant feature of the tissue. For multifocal or diffusely-distributed lesions, this grade was used for processes where between approximately 25% and 50% of the tissue in an average high-power field was involved. For focal or diffuse hyperplastic/hypoplastic/atrophic lesions, this grade was used when the affected structure or tissue had undergone between an approximately 25% to 50% increase or decrease in volume.

**Grade 4 (4+): Marked.** This corresponds to a histopathologic change that is a dominant but not an overwhelming feature of the tissue. For multifocal or diffusely-distributed lesions, this grade was used for processes where between approximately 50% and 95% of the tissue in an average high-power field was involved. For focal or diffuse hyperplastic/hypoplastic/atrophic lesions, this grade was used when the affected structure or tissue had undergone between an approximately 50% to 95% increase or decrease in volume.

**Grade 5 (5+): Severe.** This corresponds to a histopathologic change that is an overwhelming feature of the tissue. For multifocal or diffusely-distributed lesions, this grade was used for processes where greater than approximately 95% of the tissue in an average high-power field was involved. For focal or diffuse hyperplastic/hypoplastic/atrophic lesions, this grade was used when the affected structure or tissue had undergone a greater than approximately 95% increase of decrease in volume.

APPENDIX A: FIGURES AND LEGENDS


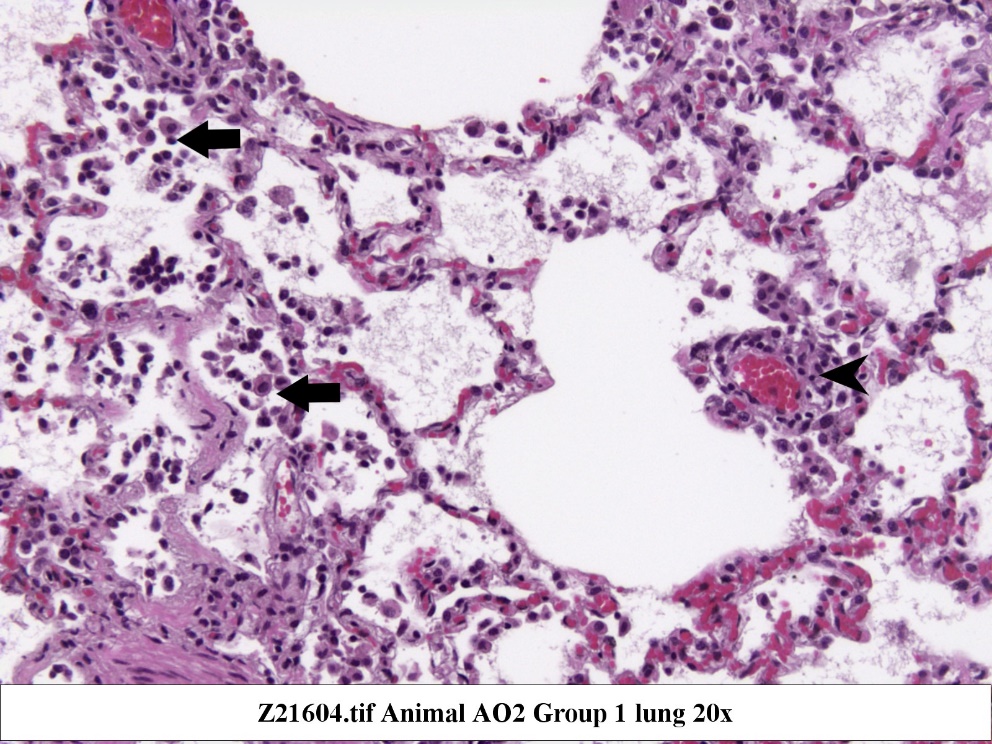


**
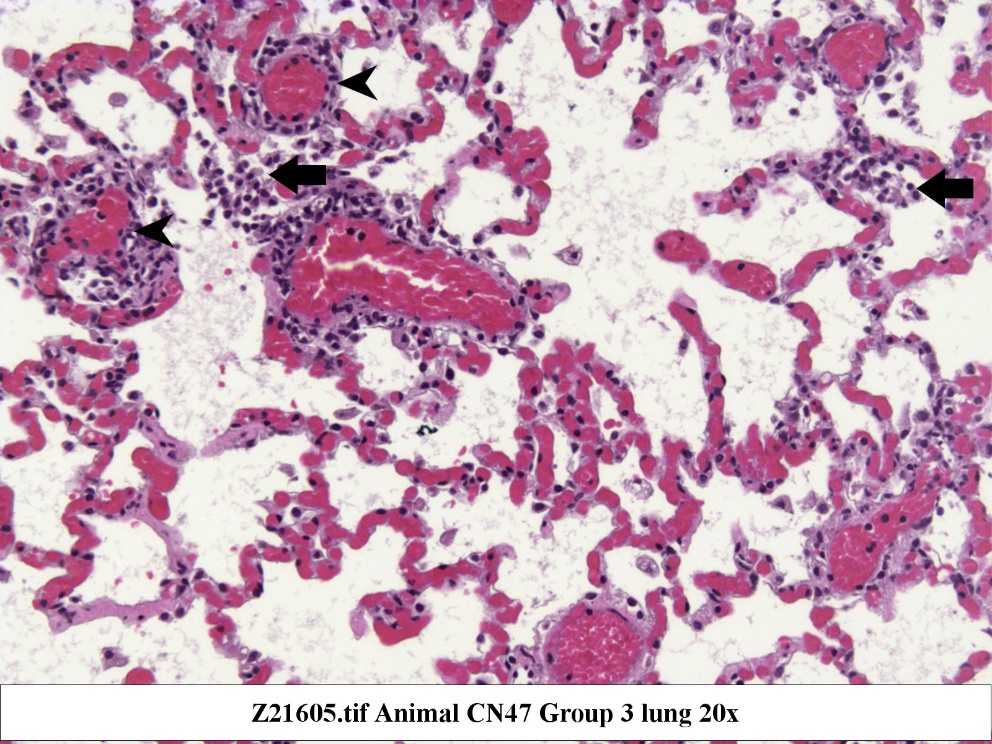
**

**Figure 1. Lung 20x.** There were no significant differences in microscopic findings in the lung of mosaic nanoparticle candidate vaccinated animals (top image) when compared to SARS-CoV-2 only challenged animals (bottom image). Mononuclear cell inflammation was observed in alveolar spaces (arrows) and perivascularly (arrowheads).
